# Short-Term Effects of Vagus Nerve Stimulation on Learning and Evoked Activity in Auditory Cortex

**DOI:** 10.1523/ENEURO.0522-20.2021

**Published:** 2021-06-17

**Authors:** Jesyin Lai, Stephen V. David

**Affiliations:** Oregon Research Hearing Center, Oregon Health and Science University, Portland, Oregon

**Keywords:** auditory, vagus nerve, plasticity, reward, learning, pupil

## Abstract

Chronic vagus nerve stimulation (VNS) has been shown to facilitate learning, but effects of acute VNS on neural coding and behavior remain less well understood. Ferrets implanted with cuff electrodes on the vagus nerve were trained by classical conditioning on an auditory tone frequency-reward association. One tone was associated with reward while another tone was not. Tone frequencies and reward associations were changed every 2 d, requiring learning of a new relationship. When tones were paired with VNS, animals consistently learned the new association within 2 d. When VNS occurred randomly between trials, learning within 2 d was unreliable. In passively listening animals, neural activity in primary auditory cortex (A1) and pupil size were recorded before and after acute VNS-tone pairing. After pairing with a neuron’s best-frequency (BF) tone, responses by a subpopulation of neurons were reduced. VNS paired with an off-BF tone or during intertrial intervals had no effect. The BF-specific reduction in neural responses after VNS remained, even after regressing out changes explained by pupil-indexed arousal. VNS induced brief dilation in the pupil, and the size of this change predicted the magnitude of persistent changes in the neural response. This interaction suggests that fluctuations in neuromodulation associated with arousal gate the long-term VNS effects on neural activity.

## Significance Statement

Vagus nerve stimulation (VNS) has been demonstrated to facilitate learning of sensory and motor behaviors. It is believed to trigger neuromodulator release that mediates cortical plasticity associated with learning. This study explores short-term VNS effects that can support long-term plasticity in the auditory cortex (A1). Just 2 d of VNS were adequate to support enhanced learning of an auditory discrimination task. Neural recordings from A1 revealed briefly pairing VNS with a neuron’s best-frequency (BF) tone reduced responses in a subpopulation of neurons. This reduction persisted even after regressing out responses explained by pupil size, a measure of global arousal. These results support a role for VNS in auditory learning and help establish VNS as a tool to facilitate neural plasticity.

## Introduction

Chronic vagus nerve stimulation (VNS) has been reported to improve learning and memory in humans (Clark et al., 1999) and rats (Clark et al., 1995). Previous studies have demonstrated that VNS during rehabilitative training improves recovery of motor function in several models of brain injury (Hays et al., 2014a,b, 2016; Khodaparast et al., 2014, 2016; Meyers et al., 2018). The therapeutic benefits of VNS during motor rehabilitation persist even after the cessation of stimulation, suggesting that VNS-induced plasticity and learning are long-term (Hays et al., 2014a; Khodaparast et al., 2016). VNS has been used in clinical therapies for epilepsy, depression and other neurologic disorders (Groves and Brown, 2005). VNS has also been reported to enhance memory and to facilitate extinction of fear conditioning in rats (Peña et al., 2013, 2014). These diverse findings suggest that VNS facilitates wide-ranging neural plasticity, which could also support learning of new auditory categories.

Although VNS has multiple clinical applications, studies investigating mechanisms by which VNS facilitates neural plasticity are limited. Recruitment of neuromodulatory activity by VNS is believed to contribute to enhanced learning (McGaugh, 1989; Engineer et al., 2015). Both the cholinergic and adrenergic systems have been implicated as playing a role. Afferent signals from VNS have been reported to activate widespread release of these neuromodulators in the brain via the nucleus tractus solitarius, the locus coeruleus (LC) and nucleus basalis (NB; Dorr and Debonnel, 2006; Engineer et al., 2013).

Most previous studies of VNS-mediated plasticity in the auditory system have measured plasticity following chronic VNS, e.g., 300 times/d for 20 d (Engineer et al., 2011, 2015, 2017; Shetake et al., 2012; Borland et al., 2019). Here, short-term effects of VNS on auditory learning and cortical plasticity were explored. To study behavioral effects of VNS, a paradigm was developed to measure reward association learning over 200–500 trials (1–2 d). Ferrets were trained by classical conditioning to discriminate between rewarded (conditioned stimulus positive; CS+) and non-rewarded (CS–) tones, and the rate of learning was compared with and without VNS. To measure effects of VNS on cortical activity, single-unit and multiunit neural activity was recorded in primary auditory cortex (A1) of passively listening animals. In subsequent neurophysiological recordings, VNS was paired with tone stimuli similar to those used for behavior. To determine the interaction between VNS, arousal and neurophysiological activity, pupil size, a measure of global arousal, was recorded during the passive stimulation experiments. Linear regression was used to dissociate effects of pupil-indexed arousal from A1 plasticity following VNS.

## Materials and Methods

### Ethics statement

All procedures were performed in accordance with the Oregon Health and Science University Institutional Animal Care and Use Committee and conform to standards of the Association for Assessment and Accreditation of Laboratory Animal Care (AAALAC).

### Animals

**Figure 1.**
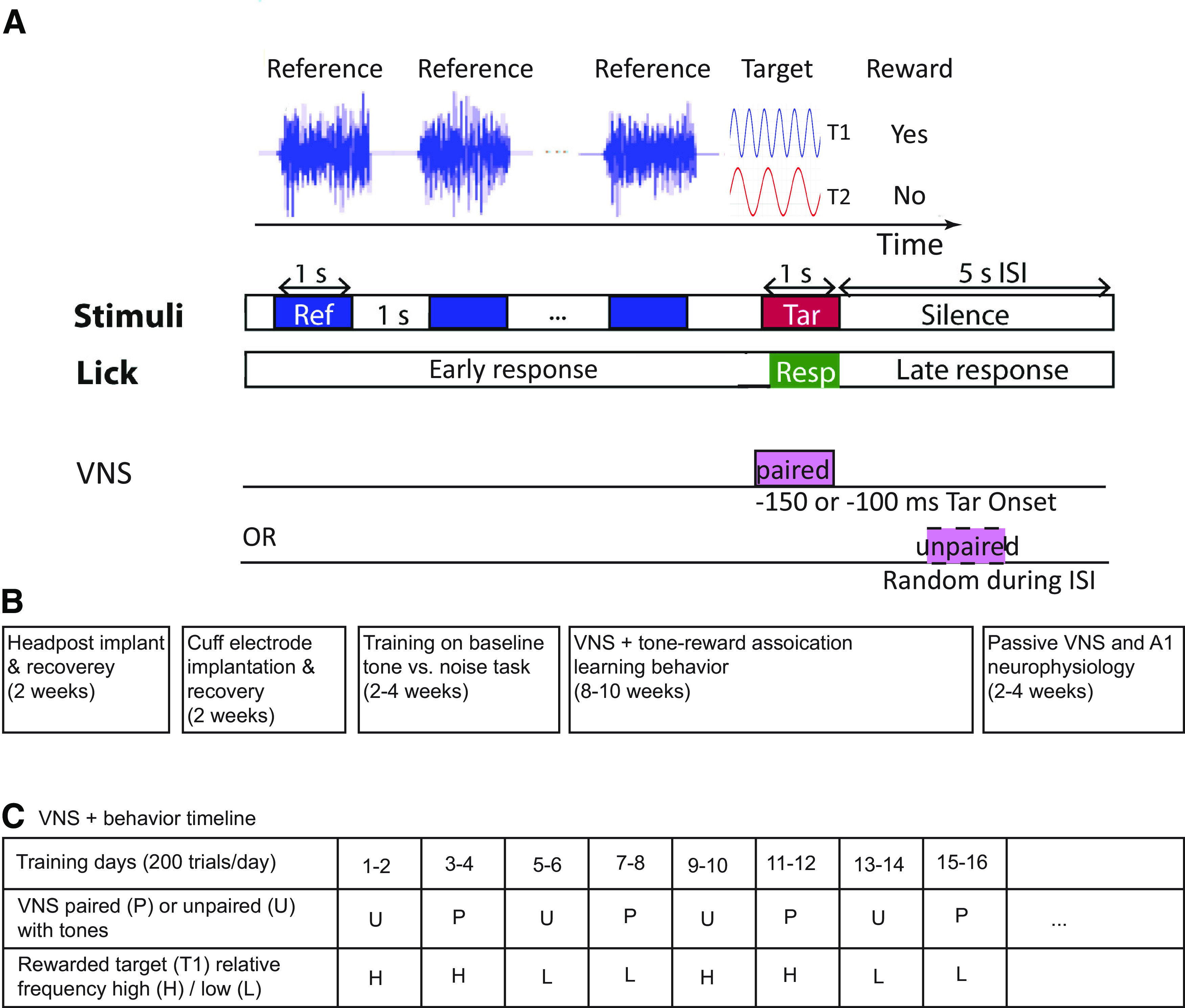
Classical conditioning behavior and experimental timelines. ***A***, Animals were trained using classical conditioning to associate one target tone (T1) with a reward and another target tone (T2) with no reward. T1 and T2 were changed every 2 d (200–250 trials/d), typically after target-reward associations were learned. During these 2 d of training, T1 and T2 were either paired with VNS (1-s duration, 30 Hz, 200-μs biphasic pulses, 0.4–2 mA, VNS onset 100 or 150 ms before T1/T2 onset) or unpaired with VNS (occurring randomly during the interstimulus interval, ISI). One or more licks during target presentation (0.2–1.15 s after tone onset) were considered a response to the target sound in anticipation of a reward. ***B***, Timeline reporting the broad sequence of surgeries, behavioral training, and neurophysiological recordings for each animal. ***C***, Table showing an example of behavioral training sessions for tone-reward association tasks across multiple days. The table includes different transitions for paired (P) and unpaired (U) VNS as well as whether T1 was the higher (H) or lower (L) tone. The sequences of these transitions were shuffled among different animals. Animals were trained for several weeks, two to three 2-d blocks per week.

Three young adult male ferrets (animals P, S, and N, less than nine months) were obtained from an animal supplier (Marshall Farms). Ferrets were used in this study because they have broad hearing frequency range that overlaps with that of humans. Moreover, ferrets are relatively easy to train and have an established repertoire of auditory behaviors (Fritz et al., 2003; David et al., 2012). The zeitgeber time (ZT) of the animal facility was ZT0 = 6 A.M. and ZT12 = 6 P.M. Before experiments, animals were implanted with a steel post for head fixation and to expose a portion of the skull for access to auditory cortex. Anesthesia was induced using ketamine (35 mg/kg, i.m.) and xylazine (5 mg/kg, i.m.) and maintained with isoflurane (0.5–2%). A warmed saline solution (10 ml) was given to the animals to prevent dehydration. Anesthesia depth was monitored by heart rate, respiration rate and blood oxygen percentage. Under sterile conditions, the headpost was mounted to the skull using dental acrylic (AM Systems) or Charisma composite, which bonded to the skull and to a set of stainless-steel screws embedded in the bone. After the surgery, animals were treated with prophylactic antibiotics (Baytril, 100 mg/ml, s.c.) and analgesics (buprenorphine, 0.02 mg/kg, s.c.) under the supervision of the university veterinary staff. The wound was cleaned and bandaged during a two-week recovery period. After recovery, each ferret was gradually acclimated to head fixation using a custom stereotaxic apparatus in a Plexiglas tube. Habituation sessions initially lasted for 5 min and increased by increments of 5–10 min daily until the ferret rested comfortably in the tube for at least 1 h. Timelines summarizing the sequence of surgeries, behavioral training, and neurophysiological recordings are reported in Figure 1*B*,*C*.

### Vagus nerve implant surgery

After acclimation to head fixation, each animal was implanted with a tripolar cuff electrode (Cortec or Microprobes) around the left cervical vagus nerve (Engineer et al., 2011), using a protocol adapted from work in rats (Lu et al., 2018). Since the right vagus nerve innervates the sinoatrial node, VNS on the right nerve may negatively impact heart rate (Johnson and Wilson, 2018). As a result, we chose to perform VNS on the left vagus nerve. Induction and other aspects of the surgery were the same as for the headpost implant.

Animals were placed in a supine position for implantation of the electrode cuff. Lidocaine (2 mg/kg, s.c.) was injected in the neck at the incision site, and the left cervical vagus nerve was exposed through blunt dissection of the neck and detachment from the carotid artery. The cuff was secured around the nerve, and leads from the electrode were tunneled subcutaneously to the headpost. They were then secured to the headpost implant with acrylic.

Efficacy of VNS was confirmed by observation of a heart rate drop while stimulating the nerve through the electrode leads (AM Systems 2100). Upon confirmation that the cuff was providing stimulation, the neck was sutured closed and Bacitracin antibiotic cream was applied to incision sites on the neck and head. Animals were given Baytril (10 mg/kg, s.c.) and buprenorphine (0.02 mg/kg, s.c.) for 2 d after surgery.

### Validation of cuff electrode function

To confirm function of the cuff electrode following implantation, effects of VNS on heart rate (under anesthesia) and pupil size (awake, passive condition) were assessed separately and on different days. For heart rate measurement, animals were anesthetized with ketamine (5 mg/kg, i.m.) and dexmedetomidine (0.05 mg/kg, i.m.). Atropine (0.05 mg/kg, i.m.) was injected to prevent bradycardia. Without atropine, heart rate fluctuation was observed, which sometimes masked VNS effects. Animals’ heart rate was compared immediately before and after delivery of current to the cuff (0.1–2 mA, 200-μs biphasic pulses at 30 Hz, 3–5 s in duration), and a drop in heart rate following VNS indicated effective stimulation. For pupil size measurement, animals were awake and head-fixed but not behaving. Pupillometry was obtained by infrared video before and after VNS, as described below.

### Pupillometry

During VNS and neurophysiological recordings, the pupil in one eye was recorded using an open-source video camera (Adafruit TTL Serial Camera) fitted with a macro lens (M12 Lenses PT-2514BMP, 25 mm). The camera was placed 10 cm from the animal’s eye. To improve contrast, the imaged eye was illuminated by a bank of infrared LEDs. Visible light was provided at constant intensity using a ring light (AmScope LED-144S), adjusted so that a maximum dynamic range of pupil size could be measured.

In early pupillometry datasets, pupil size was measured using custom MATLAB code (Schwartz et al., 2020). For later recordings, a deep neural network (DNN) was trained using Python to locate the pupil in each video frame and fit an ellipse to the pupil boundary. The algorithm was trained on video frames that were labeled using the methods in Schwartz et al. (2020). After training, the DNN performed well on novel video frames from new animals, producing results consistent with the MATLAB method. If the fit quality was poor, additional frames from the dataset were labeled, and the model was retrained to obtain a better fit quality. The code is available at https://github.com/LBHB/nems_db.

To remove blink artifacts, rapid and transient changes in pupil size were identified (McGinley et al., 2015). The derivative of the pupil trace was taken and bins with derivatives >6 SDs from the mean were marked. Blinks were identified within these bins by screening for decreases in pupil size followed by increases. Data during a 6-s period surrounding the blink were removed from the trace and replaced by a linear interpolation of the pupil size immediately before and after the blink.

Pupil size was defined as the length of the minor axis (in pixels) of the fit ellipse. Frame rate of the cameras varied between 10 and 30 frames/s. A timestamp was recorded at the start and end of each trial. Pupil measurements were aligned and interpolated to match the simultaneously recorded neural data. This procedure ensured that the two data streams (video and neural recording) remained synchronized throughout each recording, even with occasional dropped video frames. Pupil data were shifted by 750 ms relative to spike times to account for the lagged relationship between changes in pupil size and neural activity in auditory cortex (McGinley et al., 2015).

### Target-reward association task

Following recovery from cuff implant surgery, the animals were trained on a tone frequency-reward association using classical conditioning. On each behavioral trial, a pure tone target (T1, 1 s, 60-dB SPL) was presented at a random position in a sequence of broadband noise distractor sounds [temporally orthogonal ripple combinations (TORCs; Klein et al., 2000); 1-s duration, 1-s interstimulus interval; Fig. 1*A*]. Frequencies ranging over 0.2–15 kHz were used as targets because these frequencies fall within the hearing range of both humans and ferrets. During initial training, target frequency was changed often to prevent overtraining on a specific frequency. A reward (0.8- to 1.5-ml Ensure) was delivered immediately after target offset. Liquid reward delivery was controlled electronically with a solenoid valve. Licking activity was monitored by a piezo resistor attached to the lick spout. Licks after tone onset but before reward delivery indicated anticipation of the reward and were interpreted as evidence for learning of the reward association. Trial sounds and reward delivery proceeded independently of whether animals responded to the tones or distractors.

When animals learned to associate T1 with a liquid reward, another tone (T2, frequency two to three octaves away from T1) was presented on randomly interleaved trials but with no reward. In classical conditioning protocols, T1 and T2 are referred to as conditioned stimuli, CS+ and CS–, respectively, and the liquid reward is referred to as an unconditioned stimulus (US). Licking is referred to as the unconditioned response (UR). If associative learning occurs, the UR is evoked by the CS+, before the US−. (Menda et al., 2011). In our pilot studies, animals were sometimes able to learn a new target-reward association in 2 d without VNS pairing. Training on the same association for longer periods made learning of new associations, especially reversals, difficult. Therefore, the frequencies of T1 and T2 were changed every 2 d (200–250 trials/d) in subsequent training sessions (Fig. 1*C*). This also served to prevent animals from assuming a specific frequency tone was always the CS+ or CS–.

**Figure 2.**
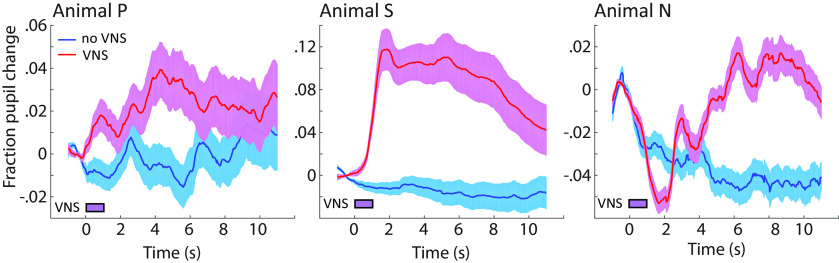
Time course of change in pupil diameter following VNS in silence. Fraction change was computed by aligning each trial to VNS onset and normalizing relative to the 1-s period before VNS. Control (no VNS) trials were interleaved with identical timing but without VNS. Animals P, S, N reached maximal dilation at ∼4.5, ∼6, and ∼7 s, respectively. Pupil dilation was significantly increased during and/or after VNS for all animals (P: *T *=* *2.3, *p *=* *0.026; S: *T *=* *6.4, *p *<* *0.0001; N: *T *=* *2.4, *p *=* *0.018, *t* test) and reached its maximum within 4.5–7 s. Shading indicates SEM.

After animals demonstrated an ability to learn new target-reward associations, VNS was introduced during behavior. Two conditions were tested, paired and unpaired VNS-tone. In the paired condition, both T1 and T2 were paired with VNS (1-s duration, 30 Hz, 200-μs biphasic pulses, 0.4–2 mA, starting 100 or 150 ms before T1/T2 onset). The temporal offset was chosen based on effective protocols reported previously for cortical plasticity following stimulation of vagus nerve (Engineer et al., 2011; Buell et al., 2019) and basal forebrain (Kilgard and Merzenich, 1998). VNS at 100 ms before T1/T2 onset was used for animals P and S, and VNS at 150 ms before T1/T2 onset was used for animal N. Given the small number of animals, it is not possible to determine whether the timing difference impacted plasticity. The relatively long delay in pupil dilation following VNS (Fig. 2) may reflect the slow dynamics of pupil muscles rather than an extended period of neuromodulatory release (Mathôt, 2018). In the unpaired condition, VNS occurred randomly during the intertrial interval, with a minimum of 500 ms following T1/T2 offset. Most VNS parameters were taken from Shetake et al., 2012, but the stimulation current was adjusted for each animal. Current was initially set at 0.4 mA, and it was increased in 0.2-mA steps until an effect was observed on learning. The maximal limit of the current was set at 2.0 mA to avoid causing discomfort during stimulation. The lowest effective current was determined as the level at which the animal showed higher cumulative lick rate for the rewarded tone consistently over 2 d of training, compared with the unpaired VNS-tone condition. Once this level was determined for each animal, the same current was then used in all subsequent training sessions and neurophysiological recordings (animal P: 1.5 mA, S: 2.0 mA, and N: 0.4 mA). While animals did not show any overt response to the VNS, they could have perceived it. However, if this was the case, the VNS could not provide an explicit cue for reward, since it was paired with both the rewarded and non-rewarded tones. The impedance of the cuff electrode (5–15 kΩ) was verified during training by converting the voltage required to produce the stimulation current into a resistance value. All data reported in the results were collected after the effective stimulation current was established.

To quantify animals’ learning to discriminate the rewarded (T1) and non-rewarded (T2) target tones, we calculated response rate difference (Δ*R*; Kalafut et al., 2014):

(1) $$\Delta R=R_{T1}-R_{T2}.$$

*R* is the probability of licking (yes or no lick for every 10 T1 or T2 trials) during the 0.95-s window from 0.2 s after tone onset to 0.15 s after tone offset. A value of Δ*R *>* *0 indicates that the animal preferentially responds to the rewarded tone, T1. No feedback was given in response to licks.

### Neurophysiology

Both neurophysiological and behavioral data were collected from the same animals. Neurophysiological recording was performed in awake, passively listening animals within one to two weeks after behavior data collection was completed. For neurophysiological recordings, a microcraniotomy was opened over A1. Extracellular neurophysiological activity was recorded using one to two tungsten microelectrodes (FHC) or a 64-channel electrode array (Masmanidis Lab, UCLA; Yang et al., 2020). Both the microelectrodes and array were inserted into A1 with independent motorized microdrives (Alpha-Omega EPS). Amplified (AM Systems 3600) and digitized (National Instruments) signals were stored using open-source data acquisition software (Englitz et al., 2013). Recording sites were confirmed as being in A1 based on tonotopy and relatively reliable and simple response properties (Shamma et al., 1993; Atiani et al., 2014). During the recording session, animals were observed and monitored by video camera. Acoustic stimulus presentation was controlled by custom MATLAB software (https://bitbucket.org/lbhb/baphy). Digital acoustic signals were transformed to analog (National Instruments), amplified (Crown), and delivered through a free-field speaker (Manger).

To isolate a spiking unit while positioning tungsten electrodes, a pure-tone or broadband noise probe stimulus was played periodically to search for sound-activated neurons. During recordings using 64-channel array, the probe was inserted into auditory cortex until neural activity was observed across the 1.05-mm span of recorded channels. A series of random, brief pure tones (100-ms duration at 60-dB SPL) was used to obtain the tuning curve and determine the best frequency (BF), i.e., the frequency that evoked the strongest spike rate response.

After characterizing tuning properties at a recording site, two frequencies were selected for probing the effects of VNS on tone-evoked activity. The timing of VNS relative to sound onset was matched to that used during behavior in the same animal. One tone was fixed at BF and other two to three octaves away from BF (off-BF). For the 64-channel array, the frequency that evoked the maximum response in most neurons was chosen as the BF. After sorting, when a neuron’s tuning curve felt within ±0.5 octave of the tone, the tone was considered as matching the BF of the neurons. The same tones were presented to passively listening animals in experimental blocks before, during and after a VNS session. The intertrial interval was 12 s, each tone was presented 20 times per block. In the during-VNS block, VNS was either (1) paired with BF tone; (2) paired with off-BF tone; or (3) unpaired with BF tone (VNS onset 6 s after tone onset). The electrophysiological amplifier was removed during the VNS session to prevent current leakage. Thus, spiking data were not acquired during the VNS session.

Pupillometry was performed during neurophysiological recordings, as described above. It was also performed during a subset of VNS sessions, when neurophysiological recordings were paused. The analysis of pupillometry during VNS versus plasticity following VNS focused on the subset of 71/110 auditory-tuned neurons for which a complete dataset was collected.

Neurophysiological data were processed offline to identify spike events. For tungsten electrode recordings, the raw signals were bandpass filtered at 300–6000 Hz and then a principal component analysis (PCA)-based clustering algorithm was applied to spike-threshold events (David et al., 2009). For array recordings, single-units and multiunits were sorted offline using Kilosort (Pachitariu et al., 2016). Neurons were considered isolated single units if the SD of events projected onto the spike waveform template was at least two times the noise floor, corresponding to >95% isolation of spikes. Spike interstimulus interval (ISI) was also inspected for high collision rates (<1 ms ISI), but the template projection criterion was adequate to exclude any units with large numbers of collisions.

### Evoked activity analysis

Spike rate and pupil data were binned at 30–50 Hz. Peristimulus time histogram (PSTH) responses to each tone were calculated by aligning spike activity to tone onset and averaging across repetitions. To obtain evoked activity, the mean spike rate during the 0.5-s silence (baseline spontaneous activity) preceding tone onset was subtracted from the PSTH.

Changes in the PSTH response to the BF tone (pre-VNS vs post-VNS) were measured in each of the three VNS conditions (BF-paired, off BF-paired, BF-unpaired). The PSTH was divided into four epochs: spontaneous activity (0–500 ms before tone onset), onset response (0–60 ms after tone onset), sustained response (60–1000 ms after tone onset) and offset response (0–100 ms after tone offset). Average spontaneous rate was subtracted from the other three responses. VNS-mediated changes in onset and offset responses were weak, and the results focus on changes in the sustained response.

### Isolation of pupil-related changes by linear regression

Based on the observation of pupil dilation following VNS and on reports that pupil size is correlated with neural excitability in A1 (McGinley et al., 2015; Schwartz et al., 2020) , we hypothesized that persistent changes in A1 activity could be explained by VNS-mediated modulation of pupil dilation. A control analysis was performed to dissociate changes following VNS that could and could not be explained by changes in pupil size. A linear regression model was fitted using 20-fold cross validation to predict response changes because of fluctuations in pupil size alone. This response (*r_pupil_*) was estimated using the recorded neural (*r*) and pupil (*p*) data.

(2) $$r_{pupil}=b_{1}*r+b_{2}*p+b_{3}*r*p+b_{0}.$$

The weights *b_i_* are the regression coefficients for effects of pupil size on baseline and response gain. The residual change in response that persisted after accounting for pupil changes (*r_persist_*) was obtained by subtracting *r_pupil_* from *r*. After regressing out the effects of pupil, a comparison of *r_persist_* before and after VNS was performed in the same way as the comparison of raw evoked responses before and after VNS described above. The pupil-corrected difference in spike rate was computed as post-VNS *r_persist_* minus pre-VNS *r_persist_*.

### Statistical analysis

When comparing the difference in means between independent samples from two different conditions, a *t* test was used to assess significance. For multiple pairwise comparisons of data samples, a repeated measure multivariate ANOVA (rmANOVA) was applied to obtain the statistics. In the analysis of sound-evoked activity in A1 neurons, the change in neural response was assessed with rank-sum test and the reduction or increase in mean response was assessed with sign-rank test. Statistics used in each analysis are detailed in Table 1.

**Table 1:** Statistical tests reported in the Results according to figure numbers

	Data structure	Type of test	Power
Fig. 2	Normal	*t* test	95% CI
Fig. 4*A*	Normal	*t* test	95% CI
Fig. 4*B,C*	Normal	rmANOVA	95% CI
Fig. 4*B,C*	Non-parametric	Pairwise *t* test	95% CI with Bonferroni correction
Fig. 7*A–C*	Non-parametric	Wilcoxon rank-sum test	95% CI
Fig. 7*D,E*	Non-parametric	Wilcoxon signed-rank test	95% CI
Fig. 8	Non-parametric	Wilcoxon rank-sum test	95% CI with Bonferroni correction
Fig. 9*A*	Normal	*t* test	95% CI
Fig. 9*B*	Normal (residuals)	Pearson’s *R*	95% CI

CI, confidence interval

## Results

Three ferrets (animals P, S, and N) were implanted with a cuff electrode for VNS. They were trained to perform the target-reward association task using classical conditioning (Fig. 1). Finally, single-unit neurophysiological data were recorded from the same animals before and after pairing VNS with acoustic stimuli.

### Pupil dilation following VNS

Function of the cuff electrode was validated by measuring changes in heart rate (Nearing et al., 2016; see Materials and Methods) and pupil size following VNS (Bianca and Komisaruk, 2007; Desbeaumes Jodoin et al., 2015; Fig. 2). Pupil size was compared following epochs with and without VNS, in the absence of acoustic stimulation. A significant increase in pupil diameter lasting for several seconds was observed following VNS in all three animals (P: *T *=* *2.3, *p *=* *0.026, peak 4.5 s; S: *T *=* *6.4, *p *<* *0.0001, peak 6 s; N: *T *=* *2.4, *p *=* *0.018, peak 7 s, *t* test). There were some differences in the time course of dilation between animals. The currents of VNS used in this study were calibrated to induce an identifiable learning effect. It is possible that animals differed in effect threshold, relative to their sensitivity to other VNS effects, such as pupil dilation. Alternatively, the variability in time course could reflect between-animal differences in the neural dynamics of coupling between VNS and pupil control centers or in the slow dynamics of pupil muscles.

### Tone-VNS pairing improved target-reward association learning

The animals were trained to associate a tone T1 with a reward (CS+) and T2 with no reward (CS–) by classical conditioning (Fig. 1*A*). T1 and T2 frequencies and associated reward values were changed pseudo-randomly every 2 d (200–250 trials/d; Fig. 1*C*). Paired and unpaired VNS conditions were varied so that transition probabilities were balanced. In addition, transitions between whether the lower-frequency or higher-frequency tone was rewarded were also balanced. Task conditions were changed every 2 d regardless of performance to prevent overtraining on a single target-reward association. Training was conducted over multiple sessions to cover all the different combinations of transition between VNS pairing and low versus high rewarded frequency. The order of these conditions was pseudo-randomized to control for possible long-term changes in performance. No long-term change in performance was observed across training sessions.

**Figure 3.**
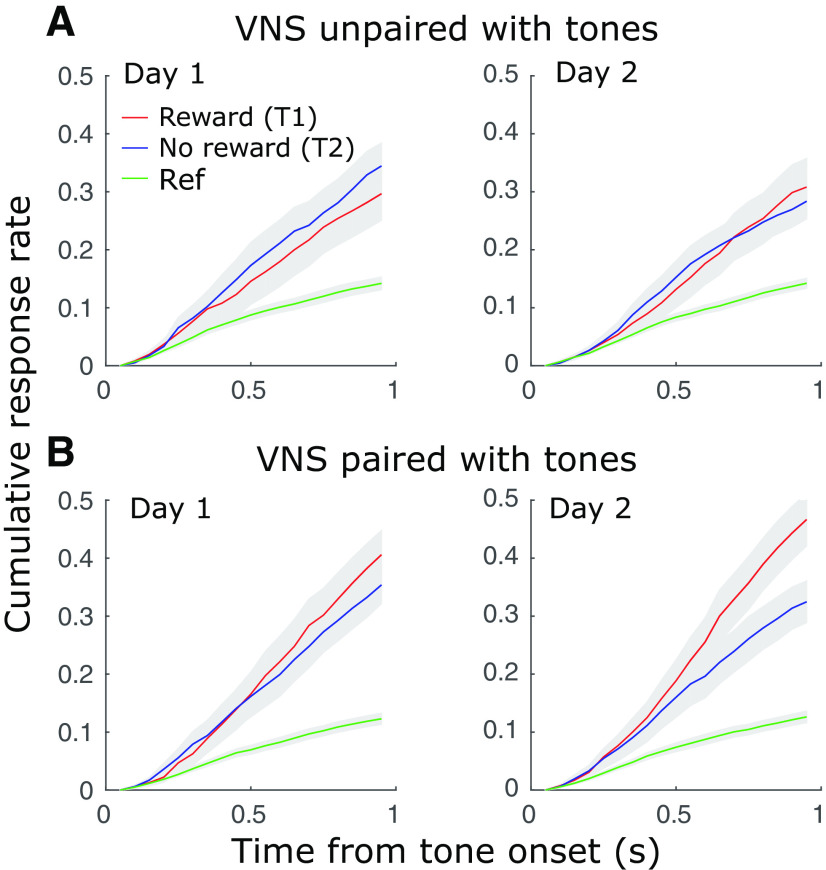
Selective responses to the rewarded tone (T1) over the non-rewarded tone (T2) increased following training. ***A***, Average cumulative response rate following sound onset across animals and training conditions (day 1 or day 2 of a tone-reward pairing, 3 animals, 66 reward association conditions total) indicates the probability that the animal licked at least once during presentation of a rewarded tone (T1), non-rewarded tone (T2), or distractor noise (Ref). There was no difference between T1 and T2 on day 1 or day 2 when VNS was unpaired with targets. ***B***, When VNS was paired with targets, cumulative response rate to T1 was higher on day 2. Reward was never presented following Ref, and these response rates were lower than for T1 and T2 in both paired and unpaired conditions.

After VNS was paired with both the rewarded (T1) and non-rewarded (T2) tones for 2 d, animals consistently responded more frequently to T1 (Fig. 3*B*). The selective response to T1 before reward delivery indicated that the animals learned the new reward association. In contrast, when VNS was unpaired with the tones (occurring between trials), animals did not show consistent evidence of learning the reward categories after the same period (Fig. 3*A*). In all cases, average response rate was consistently higher for tones than for the distractor noise, indicating an overall preference for tones over noise, the latter of which was never paired with reward.

**Figure 4.**
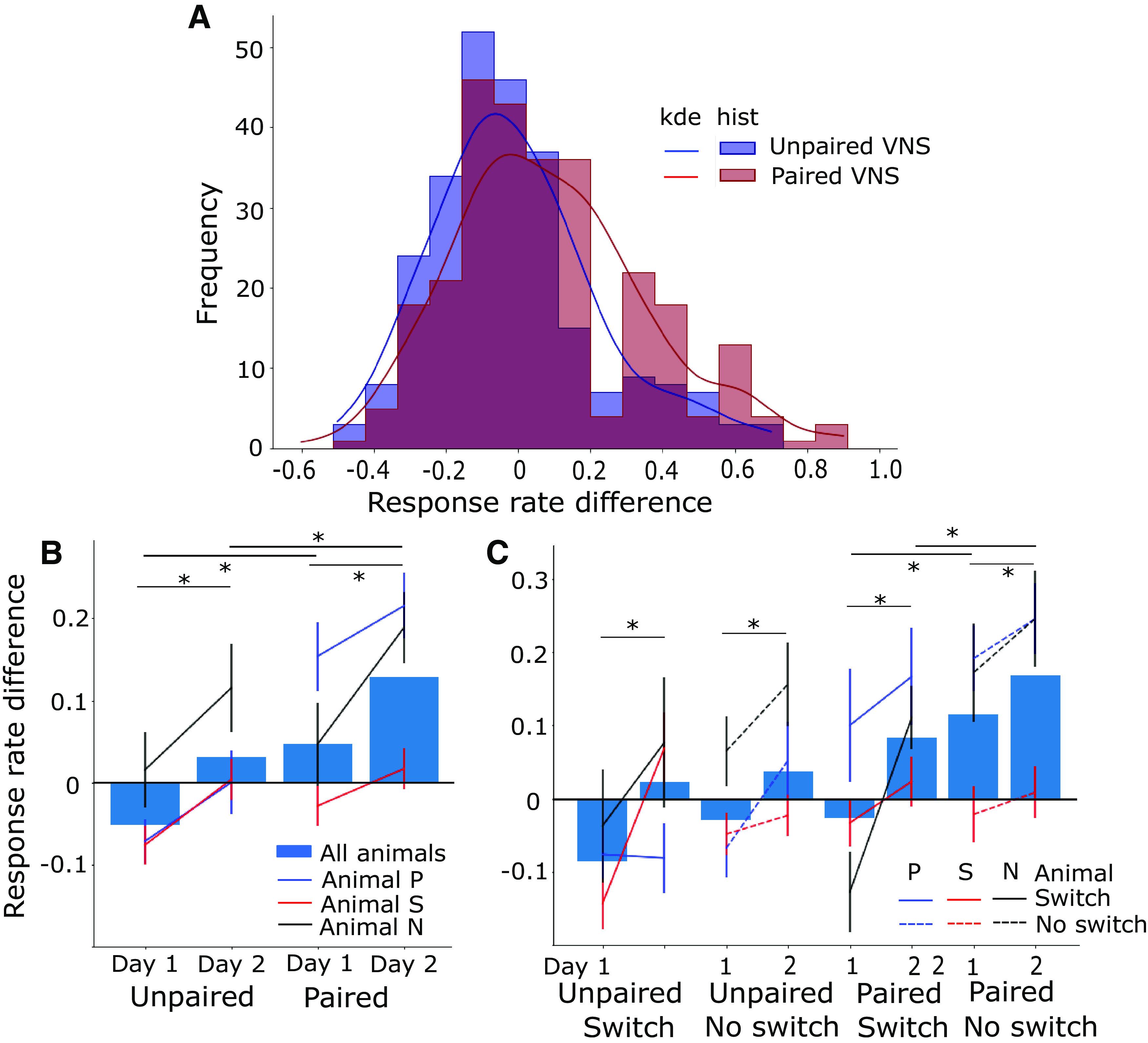
Response rate difference (Δ*R*) was larger for paired VNS-tone sessions compared with unpaired sessions. ***A***, Histogram (hist) of Δ*R* for paired VNS-tone sessions versus unpaired VNS-tone sessions. Lines denote the kernel density estimate (kde) for each histogram. ***B***, Mean Δ*R* for each day and VNS pairing condition is plotted per animal (lines) and across all animals (bars). Vertical lines indicate SEM. Animals learned to respond preferentially (Δ*R *>* *0) to the rewarded target by day 2 when VNS was paired with tone presentation (*p <* *0.05 mean difference, rmANOVA). Learning speed (slope between day 1 and day 2) was comparable for both paired and unpaired VNS-tone conditions. ***C***, Δ*R* subdivided into switch (more difficult, relative T1/T2 frequency reversed from previous session) and no-switch (less difficult, relative frequency not reversed) reward association transitions, plotted as in B. Overall Δ*R* was consistently higher when VNS was paired with tone presentation. No significant interaction was observed between task difficulty and day or VNS (rmANOVA, see text for details).

To obtain a more detailed characterization of learning across animals and days, performance was measured using the response rate difference (Δ*R*; Eq. 1), computed by subtracting the response probability to T2 (non-rewarded) from the response probability to T1 (rewarded; Kalafut et al., 2014). This quantity was measured over blocks of 20 trials within each training session. A positive Δ*R* indicated preferential responses to T1, consistent with learning that T1 preceded a reward. A value of 0 indicated equal likelihood of response to T1 or T2, and no learning of the reward association. On training days when VNS was paired with targets, Δ*R* was mostly positive and higher than for unpaired VNS-tone sessions. The kernel density estimation of Δ*R* of all paired VNS-tone sessions of all animals had a larger positive shoulder compared with that of unpaired VNS-tone sessions (Fig. 4*A*). Assessment with a *t* test showed the means of the two VNS conditions were significantly different (*T* = –4.5, *p *<* *0.05).

To summarize the learning data, response rate difference (Δ*R*) values for all paired versus unpaired VNS-target conditions were averaged, separately for day 1 versus day 2 and for each animal (Fig. 4*B*). While one animal showed above-chance performance on day 2 in the unpaired condition (animal N), mean Δ*R* across all animals was not significantly greater than zero by the end of day 2 (*T *=* *1.41, *p *=* *0.16). In contrast, the response rate difference was consistently above chance (i.e., >0) in the paired VNS-tone condition (*T *=* *5.87, *p *<* *0.05).

The breakdown between training days suggested a consistent pattern of learning reward associations over time, even when average Δ*R* was not significantly greater than zero. Multivariate ANOVA (rmANOVA) was used to test for differential effects between days. For all three animals, Δ*R* was consistently higher on day 2 compared with day 1 for both conditions (*F *=* *19.546, *p *<* *0.05). There was no interaction between VNS condition and training day (*F *=* *0.001, *p *=* *0.97).

Effects of task difficulty on Δ*R* were also measured by comparing between blocks in which the relative frequency of T1 and T2 was switched or not switched relative to the previous training sessions (Fig. 4*C*). The frequencies of T1 and T2 changed every 2 d, always maintaining two to three octaves separation and sometimes reversing which tone had higher frequency. An easier “no switch” transition occurred when a new reward association began in which the relative frequency of T1 versus T2 did not change, i.e., T1 had the higher frequency in both the current and previous conditions or had the lower frequency in both conditions. A difficult “switch” condition occurred when the relative frequencies of T1 and T2 did change. On both day 1 and day 2 of the paired VNS-tone condition, Δ*R* was lower in the more difficult condition. In all conditions (easy or difficult, paired or unpaired), Δ*R* was lower on day 1 compared with day 2 (*F *=* *18.76, *p *<* *0.05, rmANOVA). The effect of task difficulty did not significantly interact with either VNS condition (*F *=* *1.27, *p *= 0.38) or training day (*F *=* *0.03, *p *=* *0.88).

### Reduced sound-evoked activity in A1 neurons following VNS paired with BF tone

**Figure 5.**
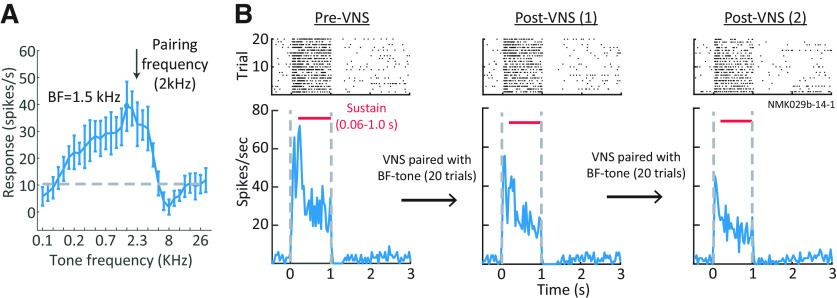
Paired VNS-tone reduced A1 neural responses. ***A***, Example tuning curve of A1 unit measured with brief, random tones. VNS was paired with a 2 kHz tone, close to the best frequency (BF, 1.5 kHz). ***B***, Raster (top) and PSTH (bottom) response of the same unit to BF tone before pairing (left), after one 20-trial session pairing VNS with the BF tone (middle), and after a second 20-trial pairing (right). The PSTH response was reduced following each pairing.

To determine effects of VNS on auditory coding, neural spiking activity was recorded in A1 of awake, passively listening animals before and after VNS. Each pre-VNS and post-VNS session consisted of 20 interleaved presentations of two tones. Across a total of 201 single-units and multiunits in A1, 110 had BF near (<0.5 octave) one of the tones. Three tone-VNS pairing conditions were tested: (1) VNS paired with BF tone; (2) VNS paired with off-BF tone; and (3) VNS during intervals between BF tone presentations. This configuration, in which VNS was paired with only a single tone, did not match the paradigm used during behavior, but it permitted measurement of the spectral specificity of plasticity effects. Spiking activity was not recorded during VNS because of the possibly of stimulation current leaking into the recording system. Tone stimulus parameters and VNS current and timing were matched to those used during behavior. PSTH responses to tones pre-VNS and post-VNS were compared. During VNS sessions, a single tone was presented 20 times, with synchronous or asynchronous VNS, as described above. Figure 5 shows the PSTH response of a neuron to the BF tone before and after VNS paired with the BF tone (tone-VNS pairing condition 1).

Since A1 excitability is correlated with pupil size (McGinley et al., 2015; Schwartz et al., 2020) and VNS can increase pupil size by itself (Fig. 2), two possible pathways were considered by which VNS might mediate changes in A1 responses. One pathway is coupled with pupil and induces changes in A1 excitability that reverse after VNS-mediated changes in pupil recover. The second pathway promotes plasticity in A1 that persists even after pupil returns to its original size.

**Figure 6.**
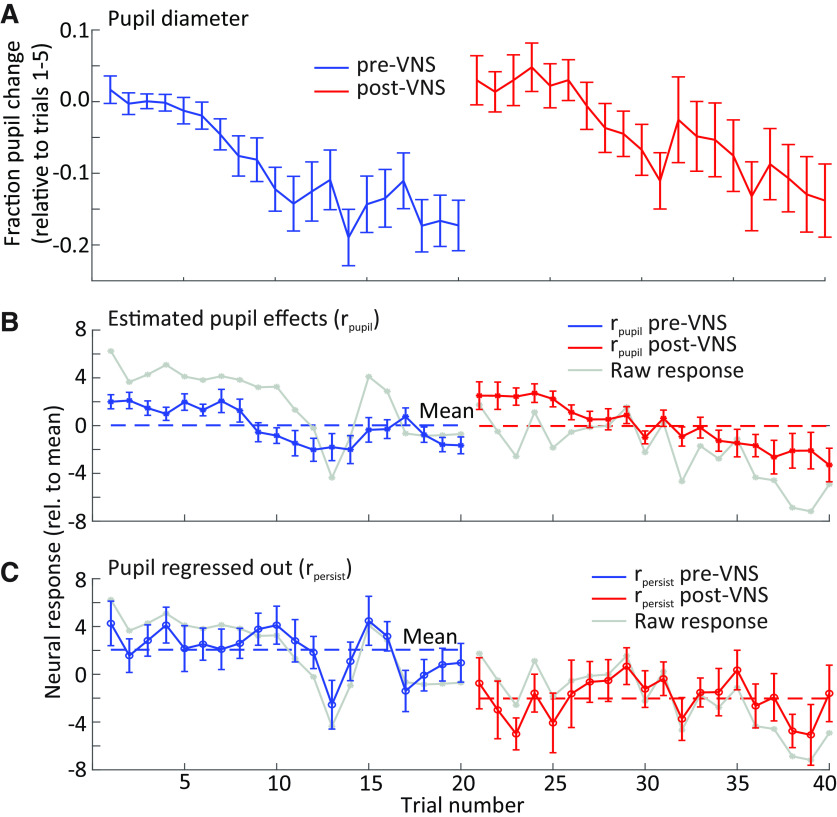
A decrease in mean neural response to best frequency (BF) tones was observed during the post-VNS session. ***A***, Fraction change in pupil diameter on each trial pre- and post-pairing of VNS with BF tone presentation, relative to baseline (mean of trials 1–5) and averaged across neurons (*n *=* *34 neurons with significant response changes after VNS). Pupil diameter decreased gradually during pre-VNS and post-VNS sessions. ***B***, ***C***, Mean change in neural response to BF tones on each trial. The mean change was separated into (***B***) the component that could be explained by pupil fluctuation (as predicted by linear regression) and (***C***) the persistent effect that could not be explained by pupil fluctuation (*n *=* *34). The latter component reflects a persistent change in response following VNS. Mean change in the raw response (gray line) is overlaid for comparison. Dashed blue and red lines indicate the mean neural response pre-VNS and post-VNS, respectively. Vertical lines indicate SEM.

To pool VNS-induced pupil fluctuations across experiments, pupil diameter measured during each experiment was normalized by the mean diameter on the first five trials of the pre-VNS session. On average, pupil diameter decreased over the course of each session (Fig. 6*A*). The large increase in pupil size at the beginning of post-VNS session probably reflected increased arousal after the experimenter entered the anechoic chamber to disconnect the stimulation system. VNS-induced changes in pupil size were smaller and shorter in duration (Fig. 2). Mean firing rate followed a similar pattern to the changes in pupil size, suggesting that changes in arousal, independent of VNS, also affects firing rate (Fig. 6*B*).

**Figure 7.**
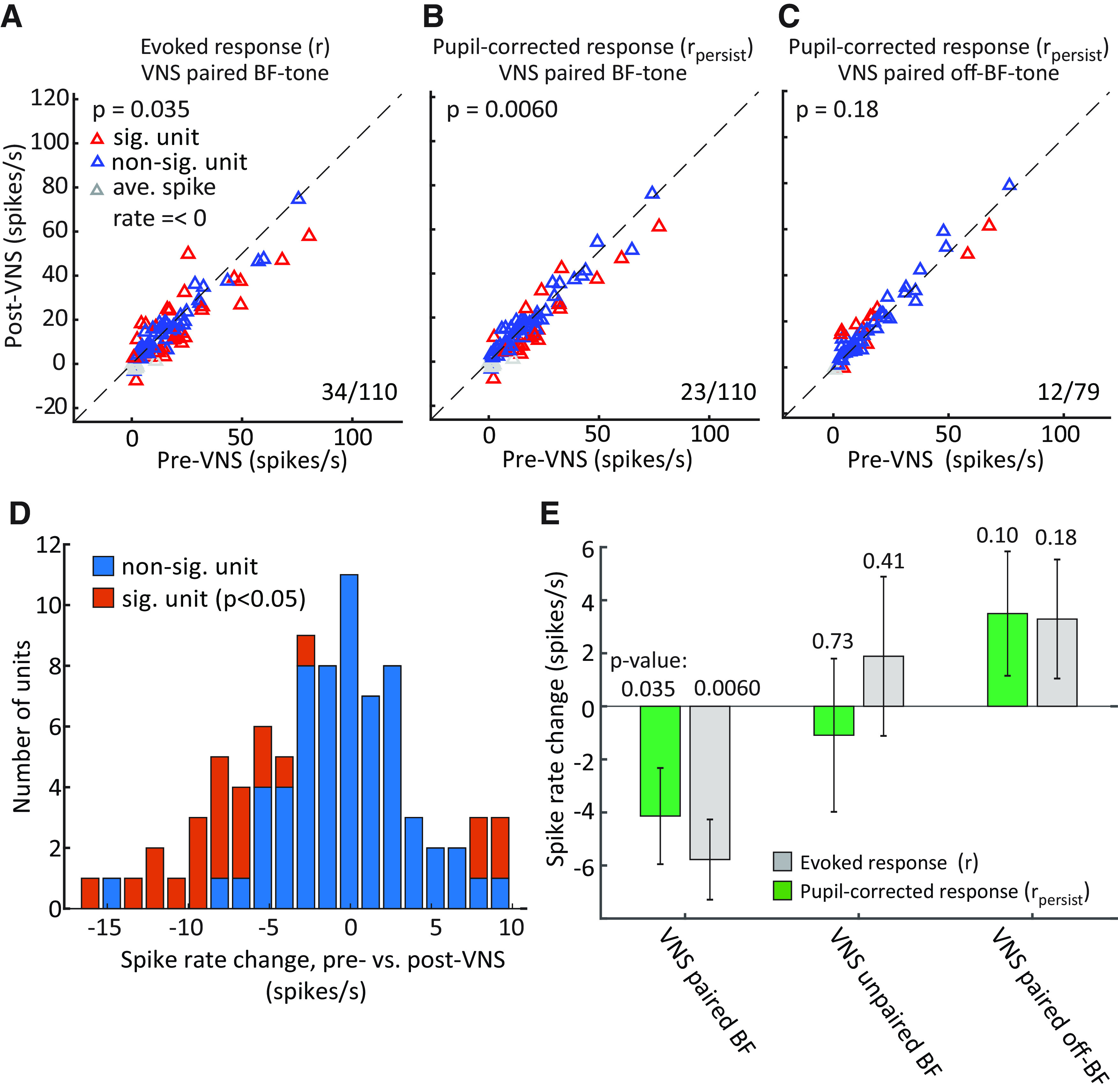
Reduced sound-evoked response in A1 after pairing VNS with best frequency (BF) tone presentation. ***A***, Scatter plot compares the sustained BF tone response for each A1 unit prepairing and postpairing of VNS with the BF tone. Red markers indicate units with a significant difference (sig. unit, *p *<* *0.05, rank-sum test). Numbers (right lower corner) indicate counts of neurons with significant difference. ***B***, Scatter plot comparing difference in sustained response after regressing out changes that can be explained by fluctuations in pupil-indexed arousal, plotted as in the left panel. The pupil-corrected response was defined $r_{persist}=r-r_{pupil}$, and *r_pupil_* was generated by the model in Equation 2. ***C***, Scatter plot of sustained response before and after pairing VNS with an off-BF tone. As in the middle panel, effects of pupil size on spike rate were removed by linear regression. ***D***, Histogram of post-VNS change in spike rate for each unit after removing changes in spike rate explained by pupil fluctuation (*r_persist_*). Most units with significant changes (red) decreased the response following VNS. Data shown were recorded in the VNS paired with BF tone condition, from panel ***A***. ***E***, Mean difference in sustained response post-VNS versus pre-VNS, for units with significant changes in auditory responses, under different VNS pairing conditions. Numbers above each bar indicate significance of the mean change (sign-rank test). Vertical lines indicate SEM.

To account for possible effects of arousal on spiking activity, linear regression was performed to measure changes in spontaneous and evoked spike rate that could be accounted for by fluctuations in pupil diameter (Eq. 2; Schwartz et al., 2020). Analysis focused on the 34/201 units with the VNS-paired tone at BF and that underwent significant plasticity following VNS (*p *<* *0.05, rank-sum test; see Fig. 7*A*). These changes were classified as pupil effects (*r_pupil_*), and residual changes, after subtracting pupil effects, were classified as persistent VNS effects (*r_persist_*). After removing pupil effects, pre-VNS and post-VNS spike rates were more stable across trials (Fig. 6*C*).

The change in the raw sustained response for each neuron was compared before and after VNS-BF pairing (Fig. 7*A*). Across the set of A1 neurons with a sustained response, 34/110 (31%) showed a significant change post-VNS (*p *<* *0.05, rank-sum test, 11/110 did not produce a sustained response in any condition). For the 34 neurons showing a difference, the mean response was significantly reduced (*p *=* *0.035, sign-rank test; Fig. 7*E*). After regressing out changes that could be attributed to pupil fluctuation, a smaller number of neurons showed significant changes post-VNS (23/110, 21%; Fig. 7*B*), but the decrease in mean response remained (*p *=* *0.006, sign-rank test; Fig. 7*D*). In contrast, the mean residual sustained response was not significantly reduced or increased when VNS was paired with an off-BF tone (Fig. 7*C*). There was also not a significant change in the mean response for the unpaired condition, when VNS occurred during the intertrial interval (results summarized in Fig. 7*E*).

A decrease in response following repeated presentations of the BF tone could reflect adaptation (Ulanovsky et al., 2003). However, the unpaired VNS condition provides a control for auditory adaptation. In this condition, VNS was not overlapped with tone presentation and instead occurred during the intertrial interval, but stimulus timing was the same. The results show no significant change in mean response, indicating that VNS outside of BF presentation has no effect on neural responses. The fact that there is no change in BF response indicates that there is no measurable adaptation when tones are presented at a rate of once per 12–13 s. Moreover, no significant change in neural responses was observed following VNS paired with off-BF tones (Fig. 7*E*), providing further evidence against effects of auditory adaptation.

**Figure 8.**
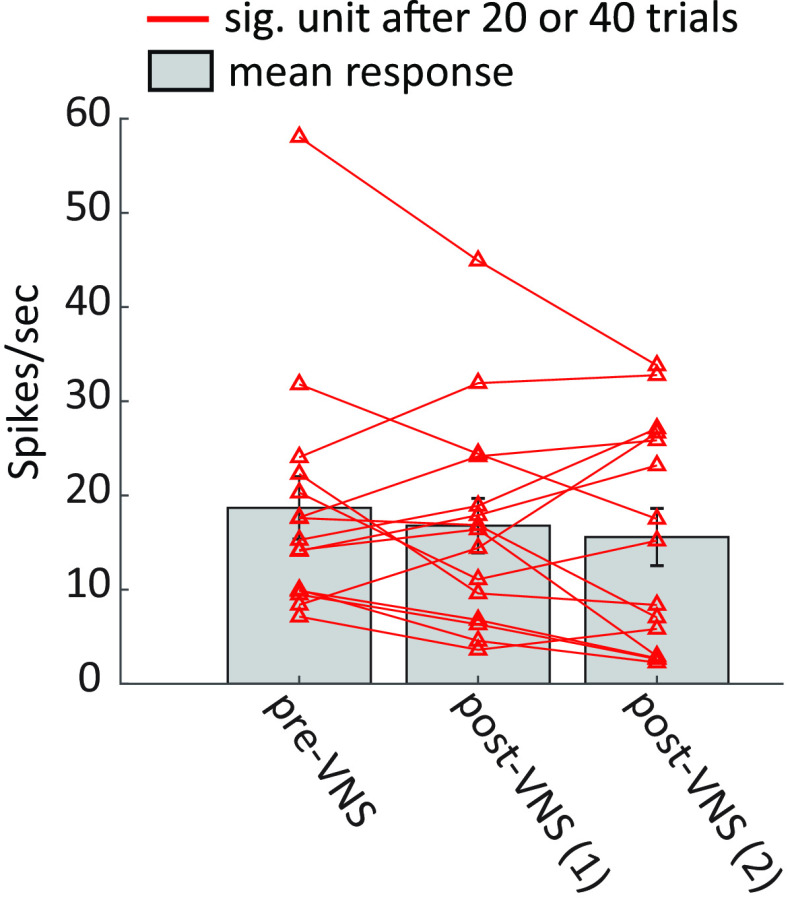
Reduced mean response for neurons with significant changes (sig. unit, *p *<* *0.05, Bonferroni-corrected rank-sum test) after first or second session of 20 trials pairing VNS with BF tone. Most neurons with significant changes show a trend toward decreased residual (pupil-corrected) response as the number of paired VNS-tone trials increased from 20 to 40. Vertical lines on bars indicate SEM.

For a subset of recordings from animal N, two sessions of 20 trials pairing VNS with BF tones were repeated. Units with significant changes after either 20 or 40 trials of VNS (*p *<* *0.05, Bonferroni-corrected rank-sum test) were selected for pre-VNS versus post-VNS comparison. Figure 8 compares pupil-corrected changes in sustained response to the BF tone. For this small subset, the decrease in response after VNS was not significant. Nonetheless, the mean response showed a trend toward greater reduction as the number of VNS trials increased.

### Pupil changes during VNS predict persistent changes post-VNS

**Figure 9.**
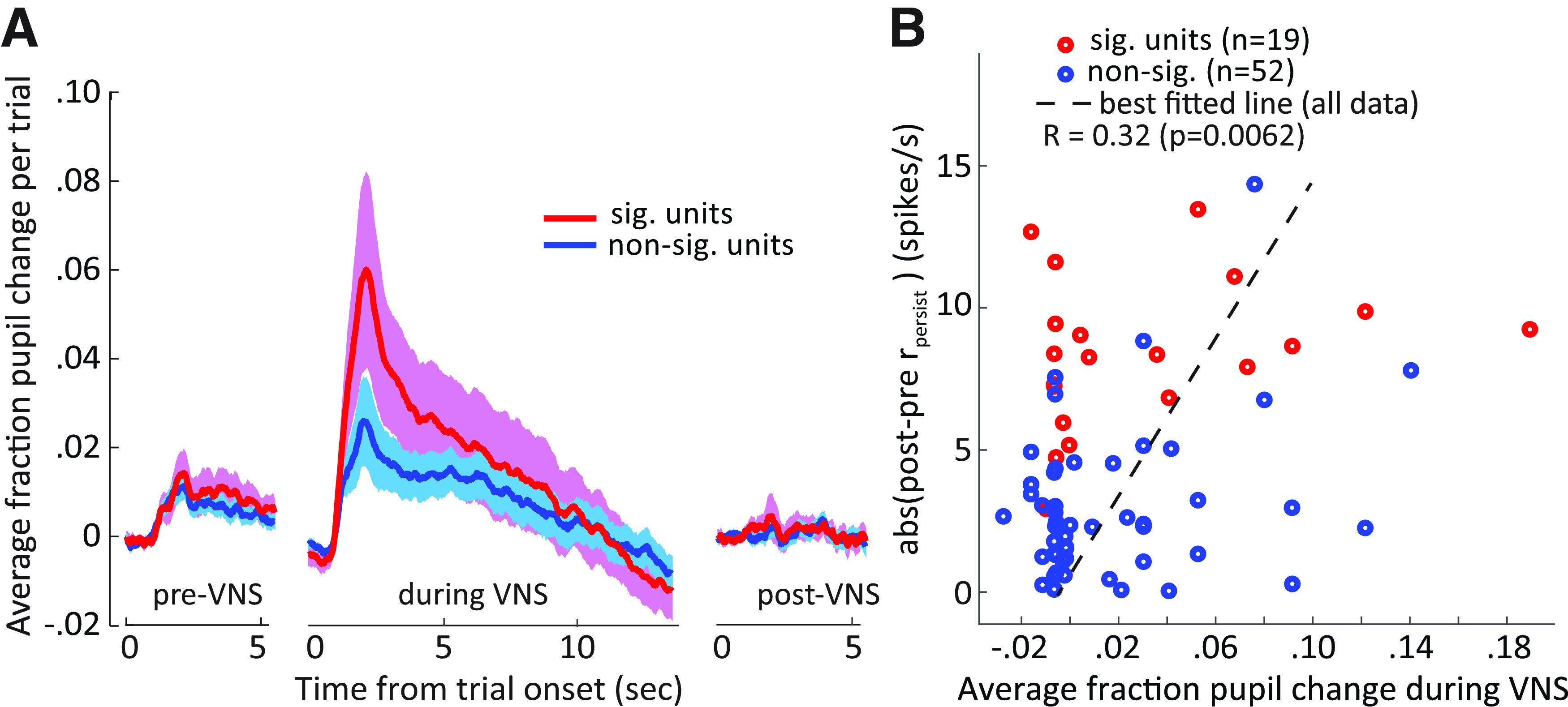
Units with significant changes between pre-VNS and post-VNS sessions were associated with larger changes in pupil size during VNS pairing. ***A***, Average change in pupil diameter on each VNS pairing trial, with change measured relative to mean pupil size during the first 0.8 s of each trial. Data shown are for VNS paired with BF tone presentation. Pupil changes were grouped by sessions in which units underwent persistent changes post-VNS (sig. units, red) or did not (non-sig. units, blue). Changes in pupil size were always large during VNS sessions, but the changes were especially large during sessions that produced persistent changes in neural responses. Shading indicates SEM. ***B***, Scatter plot compares the magnitude of persistent changes in neural response post-VNS against trial-evoked changes in pupil size during the preceding VNS (Pearson’s *R *=* *0.32, *p *=* *0.0062).

The results above demonstrate relationships between VNS and pupil size (Fig. 2) as well as between VNS and A1 excitability (Fig. 6). Changes in pupil size are associated with neuromodulatory activity (noradrenaline and acetylcholine; Reimer et al., 2016), which in turn is known to mediate cortical plasticity (Dorr and Debonnel, 2006; Engineer et al., 2013). Thus, there is a possibility that persistent changes in spiking activity following VNS could be predicted by changes in pupil size during VNS. For a subset of recordings, pupillometry was measured during VNS-BF tone pairing, allowing analysis of the relationship between pupil size during VNS and response plasticity post-VNS (*n *=* *71). Compared with pre-VNS, a larger pupil dilation was observed when VNS was paired with BF tone presentation (*T *=* *3.2, *p *=* *0.0015, *t* test, pre-VNS versus during VNS; Fig. 9*A*), consistent with the VNS-evoked dilation reported above (Fig. 2). Moreover, there was a trend toward larger pupil dilation during VNS when units showed significant persistent changes post-VNS (*T *=* *1.7, *p *=* *0.086; compare red and blue lines during VNS in Fig. 9*A*, *n *=* *19 modulated, 52 non-modulated units). Across experiments, the mean evoked dilation during paired VNS-BF tone sessions is correlated with the magnitude of the subsequent change in BF tone response (Pearson’s *R *=* *0.32, *p *=* *0.0062, *n *=* *71; Fig. 9*B*, magnitude computed as the absolute value of *r_persist_*). These results indicate that changes in pupil size during VNS predict the magnitude of persistent plasticity following VNS. This finding is consistent with the hypothesis that changes in neuromodulatory tone, reflected in pupil size, gate the long-term effects of VNS on sound-evoked activity.

## Discussion

Previous studies have shown that extended periods of pairing VNS with acoustic stimuli (300 times/d for 20 d) can induce plasticity in sound coding by A1 (Engineer et al., 2011, 2015; Borland et al., 2016; Buell et al., 2018) and auditory midbrain (Borland et al., 2019). Building on this work, the current study characterized short-term effects of VNS (200 times/d for 1–2 d) on auditory learning and stimulus-specific activity in A1. When VNS was paired with both rewarded and unrewarded tones during classical conditioning, animals responded preferentially to the rewarded tone, indicating learning of its association with the subsequent reward (Menda et al., 2011). Learning was weaker and less consistent in an unpaired VNS condition. Similar enhancements in learning were observed across difficulty conditions. These results demonstrate that short periods of VNS can have significant impact on auditory learning.

**Figure 10.**
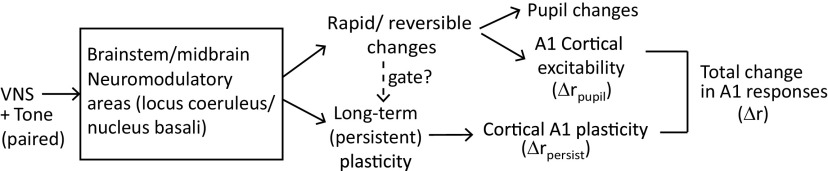
Two possible pathways by which VNS could mediate changes in A1 spiking activity. In the short-term, reversible pathway, VNS evokes changes in pupil size that are correlated with neural excitability. In the long-term, persistent pathway, which may be gated by the rapid and reversible changes, VNS produces long-term plasticity related to learning. The net change in the A1 response is the sum of short-term and long-term effects, $\Delta r=\Delta r_{pupil}+\Delta r_{persist}$.

In addition to enhanced learning on short timescales, A1 neurons in passively listening animals showed a selective reduction in response after relatively brief pairing of VNS with acoustic stimuli. This reduction persisted even after regressing out changes in spiking that could be explained by fluctuations in the pupil size, which reflect changes in global arousal (McGinley et al., 2015; Schwartz et al., 2020). Pupil dilation is observed following VNS (Bianca and Komisaruk, 2007; Desbeaumes Jodoin et al., 2015) and has been proposed as a readout of VNS efficacy (Mridha et al., 2021). Thus, these results provide evidence that changes in the activity of A1 neurons following VNS-sound pairing are mediated by two pathways (Fig. 10). First, short-term, reversible changes in excitability correlate with changes in pupil-indexed arousal (Schwartz et al., 2020). Second, persistent changes in neural activity, reflecting synaptic plasticity and learning, last longer than changes in pupil size. These effects may not emerge in cortex, as VNS can impact subcortical areas, including the inferior colliculus and thalamus (Borland et al., 2019). Thus, the effects of VNS observed in A1 could be inherited from the ascending auditory pathway. Studies using this same approach in subcortical areas can determine whether either pupil-mediated effects or VNS-mediated effects occur at these earlier processing stages.

These pathways may not be independent, as the long-term effects may be gated by the short-term fluctuations in neuromodulatory activity. Analysis of the relationship between pupil fluctuations during VNS and the persistent changes in neural responses following VNS revealed that they were in fact correlated (Fig. 9). This finding supports the idea that the rapid and reversible effects of VNS, which produce dilation in pupil diameter, positively gate long-term plasticity in A1 (Fig. 10). During neurophysiological recordings, neural responses of post-VNS were recorded within 60 min following the completion of the VNS protocol. During this period, any dynamics in VNS-related plasticity that could not be explained by dynamics of the pupil were not observed. Based on previous studies using chronic stimulation (Engineer et al., 2011; Borland et al., 2019), VNS can produced long-lasting changes. The changes observed after correcting for pupil effects in this study could be related to this long-term plasticity. Experiments that measure changes over longer time periods post-VNS can confirm this hypothesis.

### Impact of VNS on behavioral training and learning

Although research into the effects of VNS on auditory learning is limited, there is substantial evidence for positive effects of pairing VNS with rehabilitation during recovery from motor disorders (Hays et al., 2014a,b; Khodaparast et al., 2014, 2016). VNS has also been shown to reduce conditioned fear when paired with the conditioned cue during fear extinction in rats (Peña et al., 2013, 2014). Even when extinction training was delayed for two weeks after initial conditioning, animals receiving paired VNS showed significantly less fear response than sham control rats after a single day of extinction training (Peña et al., 2013). This finding is similar to the current results: animals receiving paired VNS-tone during training responded to the rewarded tone more frequently and showed significant and consistent learning effects on the second day of training (Figs. 3*B*, 4*B*,*C*). In contrast, under the unpaired VNS-tone condition, animals did not show consistent learning effects within the same training period. Animals are able to learn similar reward associations without VNS over longer periods (Kuchibhotla et al., 2017). In this study, we limited the duration of training on a single tone-reward association to 2 d to avoid overtraining on a specific rewarded tone, which can require many training sessions to reverse (Happel et al., 2014).

Previous studies of VNS in auditory learning have involved relatively simple acoustic discrimination or detection (Peña et al., 2013). Similarly, in the current study, rewarded and non-rewarded tones were separated by more than one octave. Thus, the major impact of VNS may have been on learning stimulus-reward associations rather than the discrimination of very similar stimuli. Noble et al. (2019) provide evidence that VNS enhances extinction not only for the stimulus paired with VNS but also for another CS associated with the same fear experience but not paired with VNS. Thus, it remains unclear whether VNS-sound pairing is more beneficial to learning new acoustic categories or learning reward associations with known categories.

### Importance of VNS timing and neuromodulator release in plasticity

Stimulation of vagus nerve triggers the release of neuromodulators from multiple nuclei to drive plasticity, including noradrenergic (LC), cholinergic (NB), and serotonergic (dorsal raphe nucleus) systems (Dorr and Debonnel, 2006; Nichols et al., 2011; Hulsey et al., 2019). A reduction of either noradrenergic or cholinergic signaling prevents VNS-dependent effects in the central nervous system, further suggesting that VNS engages these systems (Krahl et al., 1998; Nichols et al., 2011). Furthermore, the importance of precise timing of VNS in the induction of plasticity has also been emphasized in many studies (Engineer et al., 2011; Porter et al., 2012). Neuroplasticity is strongly influenced by the relative timing of stimuli and neuromodulator release. As a result, pairing VNS with a sensory input, for example a movement or an acoustic stimulus, improves recovery from brain injury or tinnitus by enhancing neuroplasticity in a timing-dependent manner (Hays et al., 2013; Khodaparast et al., 2014, 2016; Pruitt et al., 2016). According to the current results, animals learned reward associations faster in the paired VNS-target condition when VNS was synchronized with target tone presentation. This finding is consistent with the hypothesis that precise timing of neuromodulator release is required for the beneficial neuroplasticity associated with auditory learning.

### VNS effects on A1

There is growing evidence of parallels between effects of VNS and direct neuromodulatory stimulation. For instance, acute stimulation of NB causes widespread release of acetylcholine and can enhance the reliability of sensory coding (Goard and Dan, 2009), as well as promote learning and memory (Miasnikov et al., 2008). In their seminal work on auditory plasticity, Kilgard and Merzenich (1998) demonstrated that electrical stimulation of the cholinergic basal forebrain (NB) simultaneous to a sensory stimulus drives plasticity in auditory cortex that mimics plasticity induced by perceptual learning. Furthermore, by pairing more complex stimuli with NB stimulation, selective enhancement has been induced in A1 for a wide range of acoustic features, including sound level, temporal modulation, and frequency (Kilgard and Merzenich, 1998; Kilgard et al., 2001, 2002; Pandya et al., 2005). Noradrenergic activation may have a similar effect. Repeatedly pairing a tone with LC stimulation also induces selective plasticity for the paired frequency in A1 (Edeline et al., 2011; Glennon et al., 2019). However, NB or LC stimulation is usually performed via highly invasive deep brain stimulation, which could be harmful to the brain and thus has limited therapeutic potential. Because VNS may generate neural plasticity similar to that associated with NB stimulation (Hays et al., 2013), it may provide a safer means of stimulation that bypasses the need for deep brain stimulation.

Since afferent fibers of the vagus nerve innervate cells in the nucleus tractus solitarius, which in turn projects to LC and NB (Engineer et al., 2013; their Fig. 1), electrical stimulation of the vagus nerve should have effects similar to direct stimulation of the NB (Engineer et al., 2011, 2013). Indeed, pairing VNS with a 9 kHz tone caused a 79% increase in the number of A1 neurons with a characteristic frequency near the paired tone compared with naive control rats (Engineer et al., 2013). Moreover, repeatedly pairing VNS with rapid 15 pulse/s tone trains increased the temporal following rate of A1 neurons while pairing VNS with slow 5 pulse/s tone trains decreased the temporal following rate (Shetake et al., 2012; Engineer et al., 2013). These effects can generalize across stimuli. After pairing tone trains at rapid 15 pulse/s with VNS, A1 responses were also increased for unpaired novel speech sounds (Engineer et al., 2017). It has also been demonstrated that VNS modulates synchrony and excitability in the A1 at least in part through the activation of muscarinic acetylcholine receptors (Nichols et al., 2011).

The general observation from studies that perform chronic VNS has been of enhanced firing rate of stimulus-specific neural A1 responses (Engineer et al., 2015; Borland et al., 2019). However, the short-term effects of VNS may be different. Nichols et al. (2011) reported that shorter bouts of VNS increased and decorrelated spontaneous activity of A1 neurons and suppressed entrainment to repeated 6- to 8-Hz noise stimulation. This study recorded multi-unit activity in layers 4/5 of anesthetized rat A1 and performed 100 repetitions of VNS (500-ms train of 500-μs biphasic pulses at 30 Hz) repeated every 10 s (Nichols et al., 2011). In the current study, both single-unit and multiunit activity in A1 of awake animals were recorded. A total 20 or 40 repetitions of VNS was performed (1 s train of 200-μs biphasic pulses at 30 Hz) by repeating every 12.5 s. Despite some differences in methodology and parameters used for acute VNS, a suppression of evoked A1 responses after VNS was observed (Figs. 5, 7; similar to the observation by Nichols et al., 2011). While only a relatively small portion of recorded neurons underwent modulation because of VNS, the majority of them were suppressed. It is also important to note that some responses were enhanced and many others did not undergo plasticity after VNS. Within the BF-paired data, we were unable to identify a functional property of units that predicted the sign of plasticity or whether a unit underwent plasticity at all. It is possible that the sign of VNS-related plasticity depends on cell type or layer. A larger dataset or one that identifies cortical layer or cell type may be able to explain the variability in the sign of effects.

The explanation for why short-term VNS leads to suppressed A1 responses while long-term VNS tends to enhance responses remains unclear. Other than differences in the number of pairings between long-term and short-term VNS, the differences in A1 neural responses could also be attributed to previous pairing of VNS and rewards with sounds before neurophysiological recording. In the previous long-term VNS studies, animals only passively listened to the sounds, and they were never associated with a reward (Engineer et al., 2015; Borland et al., 2019). In addition to opposite effects on evoked activity, changes in spontaneous rate also differ. According to Borland et al. (2019), spontaneous rate was reduced in A1 of rats after long-term VNS-tone pairing, contrasting with the increase reported by Nichols et al. (2011). These differences could reflect a non-monotonic relationship between the number of VNS trials and subsequent plasticity. Alternatively, slow compensatory processes, such as changes in inhibitory network tone, could alter evoked activity over a longer time following VNS.

The observation of suppression acutely following VNS is also consistent with studies of behavior. Following engagement in some auditory tasks, average evoked activity in auditory cortex is suppressed. The specific pattern of enhancement versus suppression could depend on motor and reward contingencies of the behavior (David et al., 2012) or the balance of excitatory and inhibitory activity in the local circuit (Kuchibhotla et al., 2017). Also possibly relevant, many recordings following chronic VNS have been performed in anesthetized animals, and changes in A1 responses induced by VNS could be masked or affected by anesthesia (Cheung et al., 2001).

### Pupil dilation and gating of long-term VNS effects

Recent work has shown that neuromodulatory activity is correlated with luminance-independent changes in pupil diameter (Murphy et al., 2014; Desbeaumes Jodoin et al., 2015; Joshi et al., 2016). Spontaneous fluctuations in pupil size are correlated with changes in sensory cortical activity (McGinley et al., 2015; Vinck et al., 2015) and track rapid changes in activity of adrenergic and cholinergic axon terminals in cortex (Reimer et al., 2016). Pupil dilation has also been observed following VNS (Bianca and Komisaruk, 2007; Desbeaumes Jodoin et al., 2015) and has been proposed as a biomarker for effective stimulation (Mridha et al., 2021). Because both VNS and changes in pupil diameter are associated with fluctuations in cortical activity, the possibility that acute effects of VNS in A1 could be explained by the global changes in arousal, reflected in pupil dilation, was considered. To control for this possibility, the pupil effects were removed using linear regression. However, significant residual changes in A1 activity were observed (Fig. 7), even after the regression.

When the correlation between pupil size changes during VNS and the persistent changes in A1 activity following VNS was analyzed, a significant correlation was observed (Fig. 9). The association between the size of VNS-evoked changes in pupil and persistent changes in A1 responses suggests that plasticity in A1 is mediated by two pathways. These consist of a short-term, reversible pathway and a long-term, persistent pathway (Fig. 10). In the short-term pathway, changes in pupil size correlate with A1 excitability (Schwartz et al., 2020). In the long-term pathway, which may be gated by activation of the short-term pathway, VNS promotes persistent A1 plasticity that is related to learning improvement. Both short-term and long-term effects can be driven by VNS-mediated changes that occur downstream of VNS via neuromodulation.

This study corroborates evidence that acute stimulation of the afferent vagus nerve is associated with enhanced learning of sound-reward categories and with changes in stimulus-specific activity in A1. The relatively rapid effects of VNS on behavior may reflect enhanced learning of sound-reward associations rather than perceptual learning of sounds categories, and future studies may dissociate these effects. The observation that the magnitude of post-VNS plasticity depends on the size of evoked pupil dilation during VNS suggests that the effectiveness of VNS depends on the arousal level of the subject. Appropriately timed VNS during training may strengthen learning effects when adults acquire auditory prosthetics or new languages.

## Synthesis

Reviewing Editor: Christine Portfors, Washington State University Decisions are customarily a result of the Reviewing Editor and the peer reviewers coming together and discussing their recommendations until a consensus is reached. When revisions are invited, a fact-based synthesis statement explaining their decision and outlining what is needed to prepare a revision will be listed below. The following reviewer(s) agreed to reveal their identity: NONE.

The reviewers agree that very little is known about how vagus nerve stimulation affects learning and auditory responses in the auditory cortex and that with some revision, the study advances the field. In particular, clarification of methods and analyses would help clarify the main effects.

Reviewer 1 comments:

The manuscript “Short-term Effects of Vagus Nerve Stimulation on Learning and Evoked Activity in Auditory Cortex” (eN-NWR-0522-20) submitted for publication in eNeuro describes a set of experiments designed to test the effects of short-term vagus nerve stimulation (VNS) paired with tones on the learning of an auditory discrimination task, pupil size, and primary auditory cortex responses. The authors conclude that short-term VNS enhances learning of an auditory discrimination task. Interestingly, pairing VNS with an A1 neuron’s best frequency resulted in a reduced firing rate in some neurons. This manuscript would benefit from some methods clarification as well as greater detail about the neural responses collected in all recorded sites and the behavioral responses recorded during classical conditioning.

1. Figure 1: Was the VNS onset of -150 or -100 ms a range or was it either 100 or 150 ms? What was the rationale behind varying the timing of the onset of VNS to the onset of the sound?

2. Figure 1: In the unpaired VNS condition, was there a minimum amount of time between the offset of the sound & the onset of the VNS (could the onset of VNS occur immediately after the tone offset)?

3. Figure 1: Was there always a reward after the target tone, or was there only a reward if the ferret licked during the target presentation?

4. Figure 2,3: Typos in the legend.

5. Figure 2: What does the difference in the pupil dilation time course between animals possibly indicate?

6. Line 295: Multiple typos and unclear. Is this line supposed to be saying that the average ∆R for the unpaired condition is not significantly above chance performance after 2 days of training?

7. Line 296: Is ‘change’ supposed to be ‘chance’ (as in, chance level performance)?

8. Figure 3: I’m assuming this is an average of the 3 ferrets? Is this over a single rewarded tone/non-rewarded tone task, or over how many combinations of rewarded tone/non-rewarded tone tasks? If over multiple combinations of tasks, did the performance differ at the beginning of training (the first week) vs. the end of training (the last week)? How many weeks of training did the ferrets undergo?

9. Line 305: What counts as an ‘easy’ switch versus a ‘difficult’ switch? A frequency reversal, or a more difficult tone pair discrimination? Or both?

10. Figure 5: Was this done in the same ferrets as the behavior ferrets? If so, what was the timeline of when these experiments occurred relative to the behavior task? Same day/different day? A figure might help explain this.

11. Figure 5: How long does this reduced response last? Minutes/hours?

12. Lines 311-312: “Each pre- and post-VNS session consisted of 20 interleaved presentations of two tones.” This is how the tuning curve was collected, or this is how the VNS was paired with the tone? The methods for this are unclear.

13. Figure 6: Out of the 201 A1 recording sites, figure 6 is only plotting data from 34 of those sites. Why? What do responses from the other sites look like?

14. Figure 7D: Is this plotting the VNS paired BF-tone condition? The legend doesn’t indicate which condition this is.

15. Figure 8: Some of the neurons with significant changes show an increase in firing rate following pairing VNS with BF tone. Is there anything unique about these increased firing rate neurons? Was the tone frequency paired with VNS particularly close to the BF of the neuron?

16. Figure 9: What percent of recorded A1 neurons show this significant change in pupil size during VNS pairing? 19/201? What do the rest of the neurons look like? Why were they excluded?

Reviewer 2 comments:

When tones were paired with VNS, animals learned new tone-reward associations within 2 days; whereas random VNS and tone presentation animals did not. VNS stimulation resulted in pupil dilation, confirming prior studies. Moreover, tone-paired VNS stimulation suppressed spike rate at the neuron’s “preferred” or best frequency (BF). Neurons were separated into those that show significant spike rate change with VNS versus those that do not. Is there a rationale to explain why some neurons respond to VNS? Is it due to differences in neuron type (fast spiking vs not), cortical layer (supra vs granular), or other brain-state conditions (intrinsic brain oscillation states)?

The manuscript and analyses could be shaped to clarify the main effects of VNS. If this is done, the manuscript could advance the field in that is confirms prior studies and reports some novel effects of vagal stimulation on conditioned auditory neural and behavioral responses.

EDITS.

1. The study, “hypothesized that persistent changes in A1 activity could be explained by VNS-mediated modulation of pupil.” Edit perhaps to, “explained by VNS-mediated modulation of pupil dilation"

2. Fig. 2 legend “Dilation time course differed between animals but was always significantly increased during VNS. This would read more clearly if you computed the time delay to peak pupil dilation and show this time point on the Figure panels (e.g. Animals P, S, N reached maximal dilation at ∼4, 6, and 5 minutes, respectively). Then Edit, based on the figures, this sentence would be more correct if stated, “Pupil dilation was significantly increased for all animals and reached its maximum within 4-6 min. Also in Fig. 6 you plot the “pupil diameter” and in Fig. 2 the “pupil change”. Would be more clear if you used a similar metric for reporting these two effects. Indeed, Fig. 2 appears to be the change in pupil diameter normalized to the baseline diameter. So why not label the y-axis accordingly and explain in the legend that it is a trial average etc.

3. LL 295-296, “...response rate difference was consistently above change”. Edit, “..above chance"

4. Methods & Discussion reporting auditory learning. In the Discussion you state that “the current study characterized short-term effects of VNS on auditory learning. Yet, you are showing in Fig. 3 you’re showing average “response rates”. Are these average “correct” response rates (aka, METHODS, Delta (R) = RT1 - RT2, where T1 is the conditioning tone paired with reward and T2 is not paired with a reward) . Did you impose a time out for incorrect choices? It is not clear any “auditory learning” has happened based on what is presented or how it is reported.

5. Fig. 3, results are not clearly presented. A major point being made in the Discussion is that VNS alters auditory learning. Fig. 3 may support this but the way it is presented is not as clear as it could be. Did the animals learn and if so did they learn over fewer trials or days with VNS?

6. Fig. 5. Panel “A” , the arrow points to a position on the “tuning curve” that is >2kHz. If the paired tone frequency was 2 kHz the arrow or other label should indicate the 2kHz point on the tuning curve. In the legend, please clarify whether the spike rates illustrated in panel “B” come from the same neuron shown in panel “A”.

7. Fig. 7, Panel “E” Please refer to the compared metrics as “spike rate change” and “Normalized spike rate change” where spike rates are normalized to the change in pupil. It is unclear jargon or slang to refer to these metrics as a “Raw Response” and “Pupil regressed out”. Also, for Fig. 7D, please choose a better x-axis label “Residual VNS difference (spikes/s, post-pre)” Spike Rate Change (spikes/s) and explain how the “residual” or change is calculated in the Methods, text or legend.

8. Fig. 7 and Fig. 9, It is not clear what the physiological, behavioral or other rationale is for separating out neurons into those with and without significant change in spike rate following vagal stimulation. For example, where those units showing significant spike rate change showing fast duration spikes indicative of inhibitory neurons? Were they located in different layers of cortex? Alternatively, is the VNS related change in spike rate unreliable? What percent of the population of neurons had modulated spike rate with VNS? In Fig. 9B, you show the average pupil change elicited with VNS is weakly correlated with the spike rate change (R = 0.32). There needs to be a principled explanation for separating out units into those with significant and non-significant VNS spike rate change.

## Author Response

Reply to reviewers regarding eNeuro submission Short-term Effects of

Vagus Nerve Stimulation on Learning and Evoked Activity in Auditory Cortex

We thank the reviewers for their constructive comments. We have addressed all of them and

modified the paper accordingly and carefully revised the entire manuscript for grammatical errors

and typos. Our detailed answers follow.

Please note that reviewers’ comments are in italics, and quotes from the manuscript are indicated in blue. Line numbers refer to the marked up article file.

Reviewer 1

The manuscript “Short-term Effects of Vagus Nerve Stimulation on Learning and Evoked Activity

in Auditory Cortex” (eN-NWR-0522-20) submitted for publication in eNeuro describes a set of

experiments designed to test the effects of short-term vagus nerve stimulation (VNS) paired with

tones on the learning of an auditory discrimination task, pupil size, and primary auditory cortex

responses. The authors conclude that short-term VNS enhances learning of an auditory discrimination task. Interestingly, pairing VNS with an A1 neuron’s best frequency resulted in a reduced

firing rate in some neurons. This manuscript would benefit from some methods clarification as

well as greater detail about the neural responses collected in all recorded sites and the behavioral

responses recorded during classical conditioning.

Comment R1.1 Figure 1: Was the VNS onset of -150 or -100 ms a range or was it either 100

or 150 ms? What was the rationale behind varying the timing of the onset of VNS to the onset of

the sound?

Answer to R1.1 The VNS onset was either 100 or 150 ms before the tone onset. Previous

studies showed that stimulation of nucleus basalis (Kilgard and Merzenich 1998) or vagus nerve

(Engineer et al. 2011) anywhere between 200 ms before and 50 ms after sound onset produced

indistinguishable plasticity effects. The VNS onset times used here were within this time range.

Based on the analysis in Fig. 2, pupil changes in animal N took slightly longer time to reach its

maximum compared to animals P and S. As a result, VNS at 100 ms before T1/T2 onset was used

for animals P and S, and VNS at 150 ms before T/T2 onset was used for animal N in behavior

training and neural recording sessions.

We have clarified this aspect of task structure in the revised text (lines 187-194): This temporal

alignment was chosen based on effective protocols reported previously for cortical plasticity following stimulation of vagus nerve and basal forebrain. VNS at 100 ms before T1/T2 onset was used

for animals P and S, and VNS at 150 ms before T1/T2 onset was used for animal N. The same

VNS onset conditions were also applied in the same animal during neurophysiological recording.

Given the small number of animals, it is not possible to determine if the timing difference impacted

plasticity.

Comment R1.2 Figure 1: In the unpaired VNS condition, was there a minimum amount of time

between the offset of the sound & the onset of the VNS (could the onset of VNS occur immediately

after the tone offset)?

Answer to R1.2 In the unpaired VNS condition, there was a minimum of 500 ms between the offset of the tone and the onset of the VNS. The VNS could occur randomly within the inter-stimulus

interval but it will not occur immediately after the tone offset. We have clarified this aspect of task

structure in the revised text (lines 196-197): In the unpaired condition, VNS occurred randomly

during the inter-trial interval, with a minimum of 500 ms between tone offset and VNS onset.

1Comment R1.3 Figure 1: Was there always a reward after the target tone, or was there only a

reward if the ferret licked during the target presentation?

Answer to R1.3 For the classical conditioning paradigm used here, a reward was always delivered

after the target tone assigned to be rewarded (T1). Another tone (T2) was never paired with reward. Reward delivery following T1 offset was independent of the ferret responses, i.e., reward was

delivered regardless of whether animals licked or not. We have clarified this aspect of task structure

in the revised text (lines 163-167): Licking activity was monitored by a piezo resistor attached

to the lick spout. Licks after tone onset but before reward delivery indicated anticipation of the

reward and were interpreted as evidence for learning of the reward association. Trial sounds and

reward delivery proceeded independently of whether animals responded to the tones or distractors.

Comment R1.4 Figure 2,3: Typos in the legend.

Answer to R1.4 Thanks. Corrections have been made in the legends of Figures 2 and 3.

Comment R1.5 Figure 2: What does the difference in the pupil dilation time course between

animals possibly indicate?

Answer to R1.5 This is a good question. VNS levels were calibrated to induce an identifiable

learning effect, and it is possible that animals differed in their thresholds for a learning effect, relative to their sensitivity to other VNS effects (such as pupil dilation). Alternatively, the variability

in time course could reflect between-animal differences in the neural dynamics of coupling between

VNS and pupil control centers or in the slow dynamics of pupil muscles. In the revision, we clarify

that, while VNS consistently produced dilation, other factors could influence the magnitude and

time course of that dilation (lines 323-331): A significant increase in pupil diameter lasting for

several seconds was observed following VNS in all three animals (P: T = 2.3, p = 0.026, peak

4.5 s; S: T = 6.4, p < 0.0001, peak 6 s; N: T = 2.4, p = 0.018, peak 7 s, T-test). There were

some differences in the time course of pupil dilation between animals. The currents of VNS used

in this study were calibrated to induce an identifiable learning effect. It is possible that animals

differed in effect threshold, relative to their sensitivity to other VNS effects, such as pupil dilation.

Alternatively, the variability in time course could reflect between-animal differences in the neural

dynamics of coupling between VNS and pupil control centers or in the slow dynamics of pupil

muscles.

Comment R1.6 Line 295: Multiple typos and unclear. Is this line supposed to be saying that the

average R for the unpaired condition is not significantly above chance performance after 2 days

of training?

Answer to R1.6 Typos were corrected, thanks (lines 372-374). You are correct, the average

∆R across animals for the unpaired condition is not significantly above 0 (i.e., not above chance

performance) by the end of day 2.

Comment R1.7 Line 296: Is ‘change’ supposed to be ‘chance’ (as in, chance level performance)?

Answer to R1.7 Yes, the word ‘change’ has been changed to ‘chance’ (line 375).

Comment R1.8 Figure 3: I’m assuming this is an average of the 3 ferrets? Is this over a single

rewarded tone/non-rewarded tone task, or over how many combinations of rewarded tone/nonrewarded tone tasks? If over multiple combinations of tasks, did the performance differ at the

beginning of training (the first week) vs. the end of training (the last week)? How many weeks of

2training did the ferrets undergo?

Answer to R1.8 Indeed, Fig. 3 plotted the average of 3 ferrets and over multiple sessions (day 1

or 2) of tone reward association (rewarded tone/non-rewarded tone) task. Behavior data broken

down per animal appear in Fig. 4. These data were collected after an initial training period. The

main VNS procedures started only after animals were familiarized with the classical conditioning

task and demonstrated an ability to learn to lick preferentially after the rewarded target tones.

Relevant to this question, we also note that task difficulty varied substantially between conditions (paired/unpaired, high/low versus low/high rewarded frequency transitions). The order of

these conditions was randomized in order to control for possible long-term changes in performance.

A side-effect of this randomization is that performance varied substantially between sessions, possibly obscuring some long-term changes in performance. We clarify that we did not see any long-term

changes in performance in the reported data in the revised results (lines 342-344): The order of

these conditions was pseudo-randomized to control for possible long-term changes in performance.

No long-term changes in performance was observed at the end of all training sessions.

Comment R1.9 Line 305: What counts as an ‘easy’ switch versus a ‘difficult’ switch? A frequency

reversal, or a more difficult tone pair discrimination? Or both?

Answer to R1.9 The frequencies of the two target tones (both tones were always 2-3 octaves away)

and/or whether the high-frequency or low-frequency tone would be rewarded were changed every

2 days. For instance, when animals were trained with one set of high-frequency rewarded/lowfrequency non-rewarded tones for 2 days and then another set of high-frequency rewarded/lowfrequency non-rewarded tones for another 2 days, this is counted as an ‘easy’ switch. In contrast,

when animals were trained with one set of high-frequency rewarded/low-frequency non-rewarded

tones and then a set of low-frequency rewarded/high-frequency non-rewarded tones, this transition

is counted as a ‘difficult’ switch. The switching of target reward association was not always an exact

reversal, since we did not necessarily use the same two frequencies and swap reward association

after 2 days.

We clarify the definition of easy versus hard conditions in the revised text (lines 385-391): The

frequencies of T1 and T2 changed every 2 days, always maintaining 2-3 octaves separation and

sometimes reversing which tone had higher frequency. An easier ’8o switch’ transition occurred

when a new reward association began in which the relative frequency of T1 versus T2 did not

change, i.e., T1 had the higher frequency in both the current and previous conditions or had the

lower frequency in both conditions. A difficult ‘switch’ condition occurred when the relative frequencies of T1 and T2 did change.

Comment R1.10 Figure 5: Was this done in the same ferrets as the behavior ferrets? If so,

what was the timeline of when these experiments occurred relative to the behavior task? Same

day/different day? A figure might help explain this.

Answer to R1.10 Yes, electrophysiological and behavioral data were collected from the same

animals. Electrophysiology was performed within 1-2 weeks after behavior data collection was

completed. We indicated that data were collected from the same animals in the revised methods

(lines 222-223): Both neurophysiological and behavioral data were collected from the same animals. Neurophysiological recording was performed within 1-2 weeks after behavior data collection

was completed.

Comment R1.11 Figure 5: How long does this reduced response last? Minutes/hours?

Answer to R1.11 The post-VNS recordings lasted up to about 60 minutes following the completion of the VNS protocol. Unfortunately we did not collect data from the same neurons for a

longer period. Thus we do not have data on how long the effects last after this time. Previous

studies have shown that VNS can produce long-lasting changes, and we speculate that the changes

reported after correcting for effects of pupil are related to this long-term plasticity. Longer-term

experiments are required to confirm this hypothesis. We include this point in the Discussion (lines

515-522): During neurophysiological recordings, neural responses of post-VNS were recorded within

60 minutes following the completion of the VNS protocol. During this period, any dynamics in

VNS-related plasticity that could not be explained by dynamics of the pupil was not observed.

Based on previous studies using chronic stimulation, VNS can produced long-lasting changes. The

changes observed after correcting for pupil effects in this study could be related to this long-term

plasticity. Experiments that measure changes over longer time periods post-VNS can confirm this

hypothesis.

Comment R1.12 Lines 311-312: “Each pre- and post-VNS session consisted of 20 interleaved

presentations of two tones.” This is how the tuning curve was collected, or this is how the VNS

was paired with the tone? The methods for this are unclear.

Answer to R1.12 This sentence described how neural responses to BF and off-BF tone were

recorded during pre- and post-VNS sessions, after frequency tuning was characterized. This was

not how the tuning curve was collected. The tuning curve and properties of recording site were

characterized with brief pure tones (100 ms) at 60 dB SPL varying in frequencies randomly. The

sentence in the methods describing this process was edited for clarification (lines 241-244): A series

of random, brief pure tones (100 ms duration at 60 dB SPL) was used to obtain the tuning curve

and determine the best frequency (BF), i.e., the frequency that evoked the strongest spike rate

response.

Comment R1.13 Figure 6: Out of the 201 A1 recording sites, figure 6 is only plotting data from

34 of those sites. Why? What do responses from the other sites look like?

Answer to R1.13 Out of 201 single- and multi-units, 110 had best frequency (BF) near (< 0.5

oct) the VNS-paired tone. Among these, 34 out of 110 showed a significant change in spike rate

after VNS (p < 0.05, rank sum test, see Figure 7A). Hence, only the responses and pupil diameters

of these 34 units were included in the analysis in Fig. 6. The remaining 76 units had no significant

VNS effects, but otherwise normal auditory responses. The logic for identifying VNS-modulated

neurons was clarified in the results (lines 429-431): Analysis focused on the 34/201 units with the

VNS-paired tone at BF and that underwent significant plasticity following VNS (p < 0.05, rank

sum test, see Fig. 7A).

Comment R1.14 Figure 7D: Is this plotting the VNS paired BF-tone condition? The legend

doesn’t indicate which condition this is.

Answer to R1.14 Yes, the units plotted in Fig. 7D were recorded under the condition of VNS

pairing with the BF-tone. A sentence describing the condition was added in the legend of Fig. 7D:

Data shown were recorded in the VNS paired with BF tone condition, from panel A.

Comment R1.15 Figure 8: Some of the neurons with significant changes show an increase in

firing rate following pairing VNS with BF tone. Is there anything unique about these increased

firing rate neurons? Was the tone frequency paired with VNS particularly close to the BF of the

neuron?

Answer to R1.15 There were 4 units that showed an increase in responses post-VNS in Fig. 8.

4However, more (about 11) units showed a reduction in responses after two sessions of pairing VNS

with BF tone. Moreover, in Fig. 7B, only 4/23 modulated units showed an increase in response

post-VNS. In all cases, we paired VNS with a tone frequency close to the BF. We were unable to

fully explain why only a subset of neurons were modulated. The final analysis (Fig. 9) indicates

that the state of the animal during VNS has some impact on post-VNS plasticity. Beyond that, it

is possible that the sign of VNS-related plasticity depends on cell type or layer. A larger dataset

and/or one that identifies cell type may be able to explain the variability in the sign of effects. We

commented on this in the revised text (lines 615-621): While the majority of modulated neurons

were suppressed, it is important to note that some responses were enhanced and many others did

not undergo plasticity after VNS. Within the BF-paired data, we were unable to identify a distinctive functional property of units that predicted the sign of plasticity or whether a unit underwent

plasticity at all. It is possible that the sign of VNS-related plasticity depends on cell type or

layer. A larger dataset or one that identifies cortical layer or cell type may be able to explain the

variability in the sign of effects.

Comment R1.16 Figure 9: What percent of recorded A1 neurons show this significant change in

pupil size during VNS pairing? 19/201? What do the rest of the neurons look like? Why were they

excluded?

Answer to R1.16 There were some VNS sessions performed without pupillometry. Of the

110 units with auditory tuning, 71 had appropriate pupil data and thus we could only include

these (19/71 in this group showed post-VNS modulation). We clarify this in the revised Methods (lines 257-260): bluePupillometry was performed during neurophysiological recordings, as

described above. It was also performed in a subset of during VNS-BF tone pairing sessions, when

neurophysiological recordings were paused. The analysis of pupillometry during VNS versus plasticity following VNS focused on the subset of 71/110 auditory-tuned neurons for which a complete

dataset was collected.

We also report the criteria for establishing this subset in the results (lines 471-479): blueFor a

subset of recordings, pupillometry was measured during VNS-BF tone pairing, allowing analysis

of the relationship between pupil size during VNS and response plasticity post-VNS (n = 71).

Compared to pre-VNS, a larger pupil dilation was observed when VNS was paired with BF tone

presentation (T = 3.2, p = 0.0015, T-test, pre-VNS vs. during VNS, Fig. 9A), consistent with the

VNS-evoked dilation reported above (Fig. ??). Moreover, there was a trend toward larger pupil dilation during VNS when units showed significant persistent changes post-VNS (T = 1.7, p = 0.086,

compare red and blue lines during VNS in Fig. 9A, n = 19 modulated, 52 non-modulated units).

Reviewer 2

When tones were paired with VNS, animals learned new tone-reward associations within 2 days;

whereas random VNS and tone presentation animals did not. VNS stimulation resulted in pupil

dilation, confirming prior studies. Moreover, tone-paired VNS stimulation suppressed spike rate

at the neuron’s “preferred” or best frequency (BF). Neurons were separated into those that show

significant spike rate change with VNS versus those that do not.

Comment R2.1 Is there a rationale to explain why some neurons respond to VNS? Is it due

to differences in neuron type (fast spiking vs not), cortical layer (supra vs granular), or other

brain-state conditions (intrinsic brain oscillation states)?

Answer to R2.1 A good question. Unfortunately, we were not able to identify any distinctive

5properties of neurons undergoing plasticity during behavior. Regarding cell type, our recording

system did not allow us to identify lamina or spike width for the majority of the data. Based on

electrode insertion depths, we believe that most recordings were performed between cortical layers

1 and 5, but we are not confident of precise depth.

Our analysis of pupil changes during VNS versus post-VNS plasticity suggests that there is a

state dependency of these effects. That is, during experiments where VNS produced stronger dilation, plasticity tended to be greater. However, this dependence does not explain all the variability

across neurons. We expect that variability in cell type and micro-circuitry (e.g., the strength of

cholingeric input to a neuron) also impacts the degree of plasticity.

We have added these observations and hypotheses to the revised ms (lines 617-621): Within the

BF-paired data, we were unable to identify a distinctive functional property of units that predicted

the sign of plasticity or whether a unit underwent plasticity at all. It is possible that the sign of

VNS-related plasticity depends on cell type or layer. A larger dataset or one that identifies cortical

layer or cell type may be able to explain the variability in the sign of effects.

The manuscript and analyses could be shaped to clarify the main effects of VNS. If this is done,

the manuscript could advance the field in that is confirms prior studies and reports some novel

effects of vagal stimulation on conditioned auditory neural and behavioral responses.

EDITS.

Comment R2.2 The study, “hypothesized that persistent changes in A1 activity could be explained

by VNS-mediated modulation of pupil.” Edit perhaps to, “explained by VNS-mediated modulation

of pupil dilation"

Answer to R2.2 The sentence was edited according to the suggestion (lines 289-290).

Comment R2.3 Fig. 2 legend “Dilation time course differed between animals but was always

significantly increased during VNS. This would read more clearly if you computed the time delay

to peak pupil dilation and show this time point on the Figure panels (e.g. Animals P, S, N reached

maximal dilation at 4, 6, and 5 minutes, respectively). Then Edit, based on the figures, this

sentence would be more correct if stated, “Pupil dilation was significantly increased for all animals

and reached its maximum within 4-6 min. Also in Fig. 6 you plot the “pupil diameter” and in

Fig. 2 the “pupil change”. Would be more clear if you used a similar metric for reporting these

two effects. Indeed, Fig. 2 appears to be the change in pupil diameter normalized to the baseline

diameter. So why not label the y-axis accordingly and explain in the legend that it is a trial average

etc.

Answer to R2.3 The pupil data presented in Figures 2 and 6 were processed differently to assess

changes on different timescales. Figure 2 shows the average change in diameter per trial over

seconds (relative to diameter pre-trial), and Figure 6 shows the change in absolute diameter over

many minutes (relative to the beginning of the experiment). We agree, though, that the units of

the two analysis can be made the same. We have updated both figures to plot the fraction change

in pupil size relative to a baseline condition. For Figure 2, that is the average pupil diameter 1

sec before each VNS trial, and for Figure 6 that is the average pupil during the first 5 trials of

the experiment. We have also added more details about the timecourses plotted in Figure 2, as

suggested.

The text can be found in the revised legend of Figure 2: Timecourse of change in pupil diameter

following VNS in silence. Fraction change was computed by aligning each trial to VNS onset and

normalizing relative to the 1 s period before VNS. Control, no VNS trials were interleaved with

identical timing but without VNS. Animals P, S, N reached maximal dilation at ∼4.5, ∼6, and ∼7

6s, respectively. Pupil dilation was significantly increased during and/or after VNS for all animals

(P: T = 2.3, p = 0.026; S: T = 6.4, p < 0.0001; N: T = 2.4, p = 0.018, T-test) and reached its

maximum within 4.5-7 s. Shading indicates standard error of the mean.

The text in the Results has also been revised (lines 323-326): A significant increase in pupil

diameter lasting for several seconds was observed following VNS in all three animals (P: T = 2.3,

p = 0.026, peak 4.5 s; S: T = 6.4, p < 0.0001, peak 6 s; N: T = 2.4, p = 0.018, peak 7 s, T-test).

There were some differences in the time course of pupil dilation between animals.

Comment R2.4 LL 295-296, “...response rate difference was consistently above change”. Edit,

”..above chance"

Answer to R2.4 The sentence was edited according to suggestion (line 375).

Comment R2.5 Methods & Discussion reporting auditory learning. In the Discussion you state

that “the current study characterized short-term effects of VNS on auditory learning. Yet, you are

showing in Fig. 3 you’re showing average “response rates”. Are these average “correct” response

rates (aka, METHODS, Delta (R) = RT1 - RT2, where T1 is the conditioning tone paired with

reward and T2 is not paired with a reward) . Did you impose a time out for incorrect choices? It is

not clear any “auditory learning” has happened based on what is presented or how it is reported.

Answer to R2.5 As is standard for a classical conditioning paradigm, no time-out or other

feedback was imposed for incorrect responses or false alarms. At the beginning of day 1, animals

had no knowledge of which tone was rewarded (T1) and which tone was not (T2). A reward was

always provided immediately after the presentation of T1, and learning was measured as the rate

at which animals licked during T1 relative to during T2. Licks during T1 occurred before the

reward was delivered and thus indicated anticipation of the reward, which must be learned. One

might conceive of this approach as a positive reinforcement version of fear conditioning paradigms

widely used to study the auditory system. Learning was quantified with the normalized difference

in lick rate during T1 vs. T2, as defined in Eq. 1. We have revised the text in several places to

address these concerns:

We have clarified this aspect of task structure in the revised methods (lines 171-174): In

classical conditioning protocols, T1 and T2 are often referred to as conditioned stimuli, CS+ and

CS-, respectively, and the liquid reward is referred to as an unconditioned stimulus (US). Licking

is referred to an unconditional response (UR). If associative learning occurs, the UR is evoked by

the CS+, prior to the US.

The text introducing Fig. 3 has also been revised (lines 346-353): An analysis of average

cumulative lick rate following target onset shows that, after VNS was paired with both the the

rewarded (T1) and non-rewarded (T2) tones for two days, animals consistently responded more

frequently to the T1 (Fig. 3B). The selective response to T1 indicated that the animals learned the

new reward association. In contrast, when VNS was unpaired with the tones (occurring between

trials), animals did not show consistent evidence of learning the reward categories after the same

period (Fig. 3A).

We also reference this method for assessing learning in the Discussion (lines 492-495): When

VNS was paired with both rewarded and unrewarded tones during classical conditioning, animals responded preferentially to the rewarded tone, indicating learning of its association with the

subsequent reward.

Regarding the specific analysis of learning effects, the description of Fig. 3A has been revised

in the legend: Average cumulative response rate following sound onset across animals and training

conditions (day 1 or day 2 of a tone-reward pairing, 3 animals, 66 reward association conditions total) indicates the probability that the animal licked at least once during presentation of a rewarded

tone (T1), non-rewarded tone (T2) or distractor noise (Ref).

Finally, we added an explanation of the analysis in Fig. 4 and ∆R (lines 360-364): The quantity ∆R measured the difference in response rate between T1 versus T2 over blocks of 20 trials

within each training session. A positive ∆R indicated preferential responses to T1, consistent with

learning that T1 preceded a reward. A value of 0 indicated equal likelihood of response to T1 or

T2, and no learning of the reward association.

Comment R2.6 Fig. 3, results are not clearly presented. A major point being made in the

Discussion is that VNS alters auditory learning. Fig. 3 may support this but the way it is presented

is not as clear as it could be. Did the animals learn and if so did they learn over fewer trials or

days with VNS?

Answer to R2.6 Fig. 3 showed that animals learned to lick preferentially during the presentation

of T1 (the rewarded tone) by the end of day 2 when VNS was paired with tone presentation. In

contrast, when VNS was unpaired (occurring between trials), animals did not respond selectively

to T1 on average. Thus the mean response rates for T1 and T2 diverge in the paired condition

(panel A) but not in the unpaired condition (panel B). The more detailed breakdown of behavior

in Fig. 4 shows some evidence of learning, even in the unpaired condition, but more days of

training are likely required to achieve a consistent effect. We avoided longer periods of training on

one condition to avoid over-training, which interfered with learning of reversed associations in our

pilot study (i.e., learning in the difficult condition in this study).

We hope the changes made in response to the previous question clarify this logic. In addition,

the Discussion was edited to clarify these points (lines 532-541): animals receiving paired VNS-tone

during training responded more to the rewarded tone and showed significant learning effects on

the second day of training (Fig. 3B, Fig. 4B and C). In contrast, under the condition of unpaired

VNS-tone, animals did not learn the tone-reward association during this time period. Animals are

able to learn such reward associations over longer periods. In this study, we limited the duration

of a single tone-reward association to avoid over-training on specific reward associations, which

can be difficult to reverse (Happel et al., 2014).

Comment R2.7 Fig. 5. Panel “A” , the arrow points to a position on the “tuning curve” that is

>2kHz. If the paired tone frequency was 2 kHz the arrow or other label should indicate the 2kHz

point on the tuning curve. In the legend, please clarify whether the spike rates illustrated in panel

"B” come from the same neuron shown in panel “A”.

Answer to R2.7 We examined the figure carefully. It turns out the arrow is in fact aligned with

2kHz. The log scale is quite compressed, but a close examination of the horizontal axis indicates

that the arrow is left of the 2.3 kHz tick mark. The next tick to the left corresponds to 1.5 kHz.

The data in this figure did all come from the same unit. The legend for Fig. 5B was revised:

Raster (top) and peri-stimulus time histogram (PSTH, bottom) response of the same unit to BF

tone ...

Comment R2.8 Fig. 7, Panel “E” Please refer to the compared metrics as “spike rate change"

and “Normalized spike rate change” where spike rates are normalized to the change in pupil. It

is unclear jargon or slang to refer to these metrics as a “Raw Response” and “Pupil regressed

out”. Also, for Fig. 7D, please choose a better x-axis label “Residual VNS difference (spikes/s,

post-pre)” Spike Rate Change (spikes/s) and explain how the “residual” or change is calculated in

the Methods, text or legend.

8Answer to R2.8 We have updated the figure as suggested. We use the terms “Evoked response"

and “Pupil-corrected response", and the definitions of these measures are clarified by referencing

the Methods. We have also updated the label for the change in response in Figure 7D and E to

"Spike rate change” as suggested. The legend for Figure 7A has been revised: The pupil-corrected

response was defined rpersist = r − rpupil, and rpupil was generated by the model in equation 2.

The legend for Figure 7D has also been revised: Histogram of post-VNS change in spike rate

for each unit after removing changes in spike rate explained by pupil (rpersist). Most units with

significant changes (red) decreased the response following VNS. Data shown were recorded in the

VNS paired with BF tone condition, from panel A.

Comment R2.9 Fig. 7 and Fig. 9, It is not clear what the physiological, behavioral or other

rationale is for separating out neurons into those with and without significant change in spike rate

following vagal stimulation. For example, where those units showing significant spike rate change

showing fast duration spikes indicative of inhibitory neurons? Were they located in different layers

of cortex? Alternatively, is the VNS related change in spike rate unreliable? What percent of the

population of neurons had modulated spike rate with VNS? In Fig. 9B, you show the average pupil

change elicited with VNS is weakly correlated with the spike rate change (R = 0.32). There needs to

be a principled explanation for separating out units into those with significant and non-significant

VNS spike rate change.

Answer to R2.9 We agree that the logic for grouping neurons as modulated or not modulated is

important. Regarding Fig. 7., units with significant plasticity were defined as those that showed a

significant change in spike rate between pre- versus post-VNS spike rate. Subsequently, the mean

change of those significant units was tested to find out if the change was a significant increase

or decrease. We clarified the criteria for identifying significantly modulated neurons in the text

(lines 436-442): Across the set of A1 neurons with a sustained response, 34/110 (31 %) showed a

significant change post-VNS (p < 0.05, rank sum test, 11/110 did not produce a sustained response

in any condition). For the 34 neurons showing a difference, the mean response was significantly

reduced (p = 0.035, sign rank test, Fig. 7E). After regressing out changes that could be attributed

to pupil, a smaller number of neurons showed significant changes post-VNS (23/110, 21 %, Fig.

7B), but the decrease in mean response remained negative (p = 0.006, sign rank test, Fig. 7D).

Regarding Fig. 9, in the initial experimental design, we did not record pupil during VNS

in the passive pairing study. Thus we had to exclude a subset of units. Otherwise, this analysis

includes the entire set of BF-tuned units (VNS-modulated and non-modulated, totalling 71), as we

wanted to determine if the pupil effect could explain differences between significantly modulated

and non-modulated neurons. In Fig. 9, these units are divided into those that showed significant

post-VNS modulation after pupil correction (red vs. blue, respectively), using the same criteria

as for Fig. 7. We clarified the selection criteria in the revised text (lines 471-473): For a subset

of recordings, pupillometry was measured during VNS-BF tone pairing, allowing analysis of the

relationship between pupil size during VNS and response plasticity post-VNS (n = 71).

Thus, as the reviewer points out, we were able to identify a factor that partially explained the

magnitude of post-VNS effects, the correlation with pupil changes during VNS in Figure 9, but

we are only able to speculate as to the source of the remaining variability. Please refer to R2.1 for

revisions relevant to this question.
